# Factors predicting pain and early discontinuation of tumour necrosis factor-α-inhibitors in people with rheumatoid arthritis: results from the British society for rheumatology biologics register

**DOI:** 10.1186/s12891-016-1192-7

**Published:** 2016-08-12

**Authors:** Daniel F. McWilliams, David A. Walsh

**Affiliations:** 1 Arthritis UK Pain Centre, Academic Rheumatology, University of Nottingham, Nottingham, UK; 2 Rheumatology, Sherwood Forest Hospitals NHS Foundation Trust, Sutton-in-Ashfield, UK

**Keywords:** Rheumatoid arthritis, Biologic, Pain, National registry, Epidemiology

## Abstract

**Background:**

We examined pain levels in 2 cohorts assembled from the British Society for Rheumatology Biologics Register (BSRBR), and investigated which factors predicted Bodily Pain scores and discontinuation of TNFα-inhibitors.

**Method:**

Data were retrieved from BSRBR-RA databases for up to 1 year after commencing TNFα-inhibitors (*n* = 11995) or being treated with non-biologic therapies (*n* = 3632). Bodily Pain scores were derived from the Short Form-36 (SF36) questionnaire and norm-transformed to allow comparison with UK population averages. Discontinuation data were from physician reports. Other data, including 28-joint disease activity score (DAS28) measurements, were from clinical examination, interview, medical records and self-report questionnaires. DAS28-P was derived as the proportion of DAS28 attributed to patient-reported factors (tender joint count and visual analogue score). Missing baseline variables from both cohorts were imputed into 20 replicate datasets. Odds ratios (OR) and adjusted OR were calculated for higher than median pain within each cohort.

**Results:**

Participants reported moderate to severe pain at baseline, and pain scores remained >1SD worse than normal population standards at 1 year, even when disease activity responded to treatment. Baseline pain was associated with DAS28-P, worse physical function, worse mental health, and DAS28. After logistic regression, independent predictors of higher than median pain at follow up were baseline Bodily Pain score, higher DAS28-P, worse physical function or mental health and co-morbidities. Higher age, male gender, and higher BMI were additional independent predictors of higher pain in participants who received TNFα-inhibitors. Baseline pain was also one of the predictors of discontinuation of the first TNFα-inhibitor within 1 year, as were female gender, current smoking, co-morbidities, extra-articular manifestations and worse function.

**Conclusion:**

Pain persists in people with treated RA, even in those for whom inflammation responds to treatment. Worse pain outcomes are predicted by factors different to those typically found to predict inflammatory disease activity in other studies. Worse pain at baseline also predicts discontinuation of TNFα-inhibitors. Improved pain management should complement inflammatory disease suppression in RA.

**Electronic supplementary material:**

The online version of this article (doi:10.1186/s12891-016-1192-7) contains supplementary material, which is available to authorized users.

## Background

Treatment of rheumatoid arthritis (RA) with biologic or non-biologic disease modifying anti-rheumatic drugs (DMARDs) aims to control inflammation in order to retain functional capacity into later life. People with RA often name pain as one of the most unpleasant characteristics of their condition [1, 2]. Although patient-centred measures related to quality of life (QoL) improve during treatments that suppress inflammation [3], pain remains a problem for people with treated RA.

Inflammation is a cause of pain in RA, but other factors might also be important. Pain can persist even in people who achieve remission of inflammatory disease [4]. Previous studies have highlighted risk factors for worse pain prognosis, such as low mood [5–7] and gender [8]. We previously reported that in early RA, pain was not readily predicted by classic RA risk factors and severity measures [9]. Instead it was predicted by the proportion of 28-joint disease activity score (DAS28) contributed by the patient-reported components tender joint count (TJC) and visual analogue score (VAS-GH)), and we have proposed that a derived index, termed DAS28-P, might be related to abnormal pain processing [9, 10]. These factors that predict worse pain contrast with those that predict inflammatory disease activity or radiographic damage (recent literature reviews include [11, 12]). Predictors of pain in RA resemble risk factors that predict outcomes of other painful, chronic musculoskeletal conditions, such as low back pain [13]. Disease activity assessment, using DAS28, may be importantly confounded by pain [14, 15]. If non-inflammatory pain mechanisms inflate DAS28, then pain management strategies might be indicated rather than or in addition to escalating anti-inflammatory treatment.

In this study, we aimed to measure relationships between pain and inflammatory disease activity, and to identify baseline predictors of pain 1 year after commencing either a new biologic or undergoing non-biologic treatment in established RA. Continuation of biologic therapy should, in part, be determined by satisfactory symptomatic response and so we also measured association between pain-related factors and discontinuation of TNFα-inhibitors. We examined factors associated with baseline pain, 1 year pain and 1 year discontinuation of TNFα-inhibitors using data from 2 British Society for Rheumatology Biologics Register (BSRBR) cohorts of people with RA.

## Methods

### Data retrieval

Two biologic-naïve groups were assembled for analysis from the British Society for Rheumatology-Biologics Registry (BSRBR) national registry databases (University of Manchester, UK. Cohorts ethical approval reference number: MREC 00/8/53). The first was a hospital-based UK cohort of people initiating treatment at baseline with their first ever biologic agent. Participants were eligible for this cohort if they commenced treatment at baseline with one of 3 TNFα-inhibitors (infliximab, etanercept or adalimumab). UK national guidelines recommend that TNFα-inhibitors should be commenced after inadequate response to 2 other non-biological DMARDs [16]. Data from a total of 11995 people were supplied, of whom 92 % (11013) had baseline pain data and 68 % (8113) had 1 year pain data available and (92 %) 10949 were eligible for analysis of TNF-inhibitor discontinuation after 1 year. A second (non-biologic) cohort was hospital-based and collected by the BSRBR-RA as a referent for the BSRBR biologic cohort. Each participant was eligible if they were biologic-naïve, and were starting or receiving only non-biologic therapies at baseline. We analysed the BSRBR non-biologic cohort in its own right. The BSRBR cohorts have provided high-quality longitudinal data regarding disease activity, clinical characteristics and QoL [17]. Analyses of the BSRBR-RA cohorts have already provided data showing responses to treatment [18–20], safety [21, 22] and prevalence of co-morbidities [23].

Data from a total of 3632 people were supplied, of whom 80 % (2916) had baseline pain data and 63 % (2284) had 1 year pain data available. All participants from both cohorts satisfied 1987 American College of Rheumatology (ACR) classification criteria for RA [24]. Demographic and lifestyle data available at baseline included age, gender, smoking history and body mass index. RA disease data were seropositivity (positive or negative for rheumatoid factor); the presence of erosions (present or absent); physician-reported extra-articular RA features, which we classified as present or absent; DAS28 (TJC, swollen joint count (SJC), erythrocyte sedimentation rate (ESR), C-reactive protein (CRP), VAS-GH). The variables ESR, CRP and SJC were used as the best estimates of current inflammation. Use of each DMARD at the time of recruitment to the cohort was recorded as present, absent or data unavailable (methotrexate, sulphasalazine, hydroxychloroquine, leflunomide, azathioprine, gold salts, cyclophosphamide and ciclosporin, plus steroid use were recorded). Other related clinical data were for co-morbidities, which included physical and mental health conditions and we classified as present or absent [23]; and self-report questionnaires addressing disability Health Assessment Questionnaire (HAQ) [25, 26], or QoL (including bodily pain); Short Form-36 (SF36) (Bodily Pain, Mental Health, Vitality (fatigue) and Physical Function (disability)) [27]. Baseline DAS28 and DAS28-P (the proportion of DAS28 attributed to tender joint count and visual analogue scale) were calculated as previously reported [9, 28]. DAS28-P is a ratio of the TJC and VAS-GH, after weighting and transformation, divided by the DAS28-ESR score, and is only calculated for active RA cases due to loss of normality at low values of DAS28. The formula used was ((0.56 * √TJC) + (0.014 * VAS-GH)) / DAS28-ESR, for RA cases with DAS28-ESR ≥3.2 [9].

### Study outcomes

Norm-based SF36-Bodily Pain subscale scores at 1 year were used as the primary measure of pain outcome. This used the semi-continuous SF36-Bodily Pain scores [27], has been validated in RA populations [29], and yields a score where the mean for the UK population is 50 with a sd of 10. Use of norm-based data facilitates comparison between this study and the UK population [30]. The pain items from the SF36 were “how much bodily pain have you had during the past week?” and “During the past week, how much did pain interfere with your normal work (including both work outside the home and housework)?” SF36 version 2 was used for the data collection.

Discontinuation of TNFα-inhibitors was used as a surrogate outcome for treatment failure, and this analysis was performed independently of the prediction of pain outcome. TNFα-inhibitor use or discontinuation (plus the reason for discontinuation) was reported by each study centre at each data collection. Discontinuation was defined as absence of the initial TNFα-inhibitor treatment after baseline in a participant in the TNFα-inhibitor cohort. Switching from the initial biologic drug was taken as discontinuation, even if it was replaced by another TNFα-inhibitor. Two important reasons for discontinuation, inefficacy (IE) and adverse event (AE), were also analysed. Almost all (>99 %) of the participants that discontinued TNFα-inhibitor were using either non-biologic DMARDs or glucocorticoids at 1 year follow up. Data on any new biologics were not available for this study.

### Statistical analyses

Greater than median pain measures (at baseline or at 1 year) or 1 year discontinuation of TNF-inhibitors were the primary outcomes. Mean (standard deviation) and percentage prevalences of participant characteristics are presented. Simple univariate comparisons between groups used ANOVA (followed by pairwise testing with a Bonferroni correction), t-tests or *χ*^2^ tests. Cohen’s d statistic was calculated using the difference between means over the pooled standard deviation of both time points. Pain was stratified into bands delimited by the sd of the UK population norm (10 points) and presented graphically. ESR readings were divided into <23 for men and <30 for women for secondary analyses [31].

To attempt to address some of the biases potentially introduced by missing data, analyses using multiple imputations of 20 datasets were performed for baseline variables (age, gender, height, weight, TJC, SJC, ESR, VAS-GH, HAQ, seropositive, smoking, all SF36 variables at baseline). The follow up pain outcome variable was not imputed. Fully conditional specification methodology was used with continuous variables imputed using multiple linear regression and categorical variables using multiple logistic regression. Hosmer-Lemeshow *χ*^2^ and the Nagelkerke pseudo-R^2^ were used to assess model fit and model power respectively for complete case data. Comparisons were made between cases with complete data for all covariates and those where at least one variable would require imputation.

Imputed data were used for the primary analyses. The composite indices of DAS28 and DAS28-P were calculated from measured or imputed ESR, SJC, TJC and VAS-GH; whereas the BMI was imputed directly. Imputed continuous variables were converted into tertiles, or WHO BMI groups (<25, 25–30, ≥30 kg/m2). These are described in Additional file 1: Table S1. For associations with dichotomised outcome variables, the odds ratios (OR) and 95 % confidence intervals (CI) were calculated for the risk of above vs below median pain score (at baseline or at 1 year) or 1 year discontinuation. The univariate potential associations SF36-Bodily Pain score and discontinuation were not adjusted for multiple comparisons.

Multivariable-adjusted logistic regression models were generated, which yielded adjusted OR (aOR) and 95 % CI. The measures at baseline were used to investigate the baseline association with the pain outcome, and also to predict 1 year pain (1 year change in pain from baseline was also used so confirm findings). In order to minimise collinearity, inclusion of SF36 subscales was limited those with a priori hypothesised associations with pain; mental health, baseline pain, fatigue (SF36-vitality) and disability (SF36-physical function). DAS28 was not strongly associated with DAS28-P (TNFα-inhibitor cohort: r = 0.19; non-biologic cohort r = 0.34), and therefore this did not preclude inclusion of both within the same statistical models.

Subgroups of the cohorts at the 1 year time point were examined at the individual patient level to assess pain reported by people considered as having a good response to treatment or with low inflammation. These subgroups were either those that achieved good EULAR response [32], those satisfying remission criteria [33], those with both normal ESR levels and a swollen joint count of zero, or those who achieved CRP ≤5 g/L at 1 year.

Statistical significance was taken when *p* < 0.05 or the 95 % CI did not encompass unity.

## Results

The demographics of each cohort are shown in Table 1. The non-biologic cohort had fewer females, was older, had a shorter RA duration, and lower DAS28-P. The non-biologic cohort also displayed milder disease, with lower DAS28 and less impact on QoL than did the TNFα-inhibitor cohort (*p* < 0.003 for each comparison).

**Table 1:** Baseline characteristics of the TNFα-inhibitor and non-biologic cohorts at baseline

		TNFα-inhibitor cohort	Non-biologic cohort
		Non-imputed	Non-imputed
N=		11995	3632
Age	Years	56 (12)	60 (12)
Gender	Female	76 %	73 %
Ethnicity	White	96 %	98 %
BMI	Kg.m^−2^	27.0 (7.1)	27.4 (6.6)
Smoking	Current	22 %	24 %
	Ex-	38 %	40 %
	Never	40 %	37 %
DAS28		6.6 (1.0)	5.1 (1.3)
	TJC	16 (7)	8 (7)
	SJC	11 (6)	6 (5)
	ESR	46 (29)	35 (25)
	VAS-GH	73 (20)	55 (24)
DAS28-P		0.48 (0.07)	0.44 (0.10)
Duration of RA	Years	13 (10)	10 (10)
Serology	Positive	65 %	58 %
Erosions	Yes	63 %	46 %
Extra-articular	Yes	29 %	19 %
Comorbidity	Yes	60 %	65 %
HAQ		2.0 (0.6)	1.5 (0.8)
SF36-Vitality		33 (10)	39 (10)
SF36-Mental Health		40 (11)	45 (11)
SF36-Physical function		16 (11)	24 (14)
SF36-Bodily Pain		25 (7)	31 (9)
Steroids at baseline		44 %	23 %
DMARD at baseline	Methotrexate	58 %	65 %
	Sulphasalazine	15 %	32 %
	Azathioprine	2 %	2 %
	Leflunomide	8 %	12 %
	D-penicillamine	1 %	1 %
	Gold	2 %	5 %
	Hydroxychloroquine	10 %	15 %
	Ciclosporin	1 %	2 %
	Cyclophosphamide	0.10 %	0.10 %

The baseline characteristics of participants are shown, at baseline with and without imputation. Mean (sd) or percent are shown. The standard deviations of the 20 imputed datasets are derived from the average sd of each imputation. All SF36 scores are norm-based and may be compared to the UK mean (sd) score of 50 (10), with lower scores indicating worse health. Serology status was recorded as Rheumatoid Factor positive prior to, or at, baseline

*ESR* erythrocyte sedimentation rate, *SJC* swollen joint count, *TJC* tender joint count, *VAS-GH* visual analogue scale-general health

**Fig. 1:**
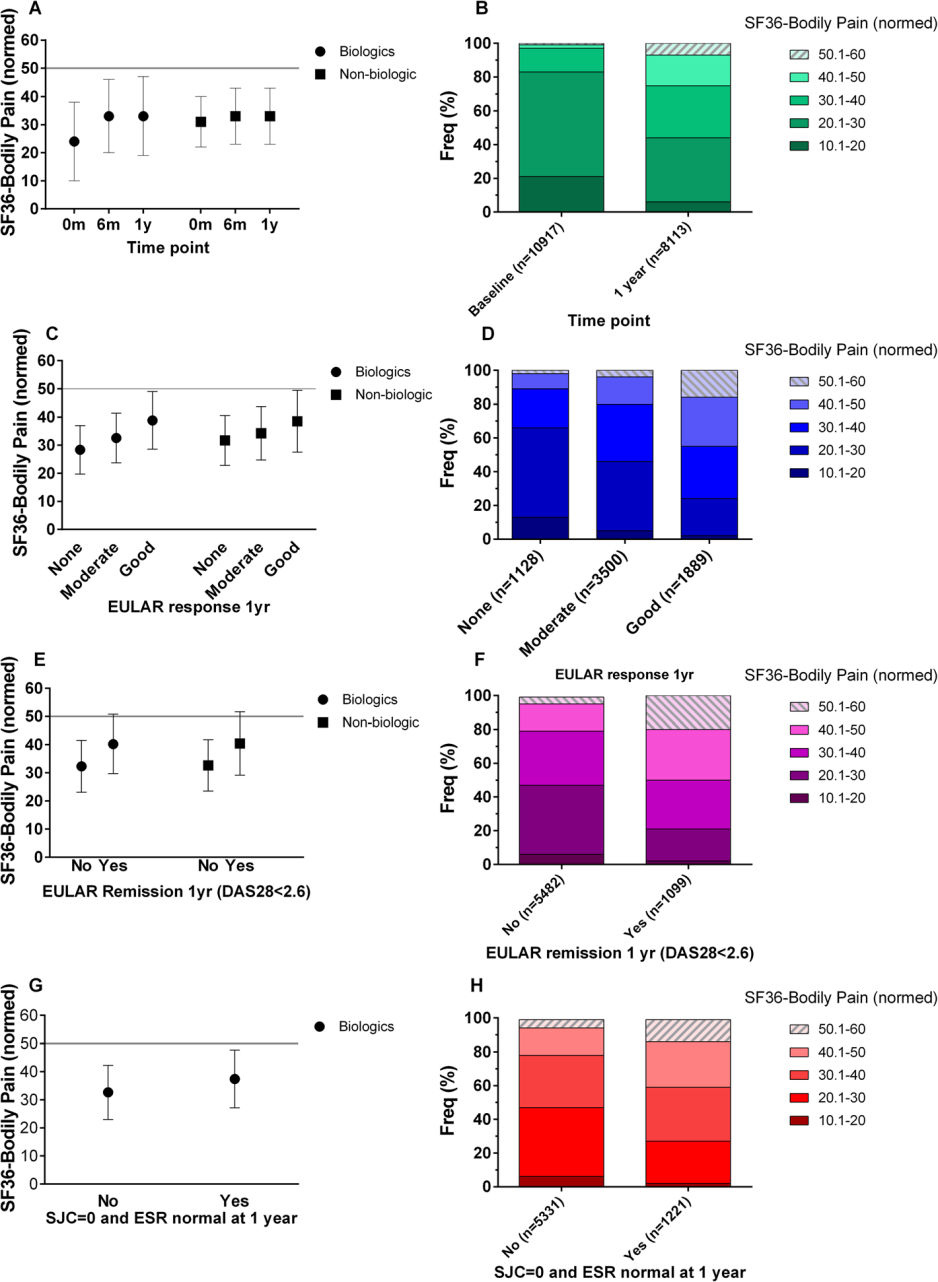
Pain up to 1 year of follow up. **a**, **c**, **e**, **g** show mean (sd) of norm-based SF36-Bodily Pain Scores using all data available at each time point. The UK population has an average norm-based score of 50 (grey, solid line), and lower scores indicate worse pain. **b**, **d**, **f**,) show the individual patient data from the TNF-inhibitor cohort demonstrating the proportion of participants with pain levels stratified by the norm-based standard deviation of 10. **a** shows mean (sd) of SF36-Bodily Pain scores from baseline to year 1. **c**, **e** and **g** show mean (sd) of norm based SF36-Bodily Pain scores at 1 year stratified by treatment response using **c** EULAR criteria; **e** DAS28 remission; and **g** normal swollen joint count (SJC = 0) with normal ESR range. Panels **b**, **d**, **f** and **h** show the stratification of individual patient data by SF36-Bodily Pain score in the TNF-inhibitor cohort at 1 year follow up. The cross-hatched, light shaded portion represents “better than UK average SF36-Bodily Pain” (≥50) and darker shaded plain portions represent increasingly worse pain at 1 year (increments of 10, which is ~1 sd for norm based SF36 scores). **b** Proportions individual patients with increasing severity of SF36-Bodily Pain scores at 1 year follow up; **d** stratified by EULAR response criteria, **f** stratified by DAS28 remission criteria, and **h** stratified into normal ESR and SJC ranges

Mean SF36-Bodily Pain scores at baseline were >2 s.d. higher than the UK general population average, indicating moderate to severe pain. The respective median self-report for TNF-inhibitor and non-biologic cohorts pain levels were ‘severe’ and ‘moderate’; and the respective interference that pain had on their normal work, including housework, was ‘quite a bit’ and ‘moderately’. Bodily Pain scores improved significantly after 6 months in both cohorts, and these improvements were maintained at 1 year (p < 0.001 for all comparisons) (Fig. 1a). The improvements and Cohens d statistic for pain improvement between baseline and year 1 was 0.91 for TNFα-inhibitor cohort and 0.04 for non-biologic cohort. After 6 months of treatment, reported pain was comparable between TNFα-inhibitor and non-biologic cohorts. Mean Bodily Pain scores in both cohorts remained >1 s.d. higher than the average for the UK population after 1 year (Fig. 1a). After 1 year, The median self-report for TNF-inhibitor and non-biologic cohorts pain levels were both ‘moderate’; and the interference that pain had on their normal work, including housework were both ‘moderately’).

Further analyses examined whether persistent pain was also evident in people in whom the inflammatory component of their disease was well controlled at the 1 year time point. In the TNFα-inhibitor cohort, 6517, 6581, 6210 and 4341 participants provided data for 1 year analysis of EULAR response; DAS28 < 2.6 remission; normal levels for both ESR and swollen joints (0/28); and measurements of CRP ≤5 g/L. Fifty four percent of participants achieved moderate, and 29 % achieved good EULAR responses. Seventeen percent achieved DAS28 < 2.6 remission. Twenty percent achieved normal levels for both ESR and swollen joints. Thirty three percent achieved CRP ≤5 g/L. Pain improved significantly after 1 year (Fig. 1a), in those with moderate and good EULAR responses (Fig. 1c), for those in DAS28 remission (Fig. 1e) and for those with normal ESR and SJC at 1 year (Fig. 1g) (*p* < 0.001 for all comparisons). Despite this improvement in pain, the SF36-Bodily Pain scores at 1 year indicated higher pain than UK population means in each of these ‘well controlled’ subgroups (Figs. 1c, e, g). Analysis of individual patient data at the 1 year time point revealed that more than half of the participants in each ‘well controlled’ subgroup had SF36-Bodily Pain scores >1 sd worse than the national average, that is a normed SF36-Bodily Pain score of less than 40 (Figs. 1d, f, h). Similar analysis was performed in the non-biologic cohort, where available data indicated that 29 % (265/905) achieved moderate, and 23 % (207/905) good EULAR response criteria, and 18 % (155/909) achieved DAS28 < 2.6 remission at 1 year. Of those with good EULAR response or those in DAS28 remission, 58 or 50 % (respectively) showed 1 year SF36-Bodily Pain scores >1 sd worse than national average (Figs. 1c and e).

After the characterisation and description of pain progression, further analysis focussed upon factors associated with pain. The variables at baseline with the greatest percentage of missing values in the TNF-inhibitor cohort were HAQ (7 %), ESR (8 %), DAS28-P (9 %), DAS28-ESR (9 %), BMI (12 %) and SF36 subscales (9 - 10 %). In the non-biologic cohort the greater percentages of missing values were found in HAQ (20 %), ESR (11 %), DAS28-ESR (12 %), DAS28-P (18 %) and SF36 subscales (19 - 20 %). In the TNFα-inhibitor and non-biologic cohorts, DAS28-ESR was higher in the complete cases than in cases with imputed 1 year values (6.6 vs 6.4 and 5.3 vs 4.4 respectively), and SF36-Bodily Pain scores indicated worse pain (25 vs 26 and 31 vs 33 respectively). Baseline SF36-Bodily Pain scores were analysed for all available data and were associated with many baseline factors in each cohort (Table 2). The baseline characteristics that predicted median SF36-Bodily Pain scores indicating worse pain at 1 year were similar between TNFα-inhibitor and non-biologic cohorts in univariate analyses (Table 3). Baseline characteristics of older age, higher BMI, current smoking, co-morbidities, DAS28, DAS28-P, and worse mental health, vitality, physical function and pain were positive predictors of SF36-Bodily Pain scores indicating worse pain at 1 year in both cohorts (Table 3). SF36-Bodily Pain scores indicating worse pain at 1 year were also predicted by male gender and extra-articular disease at baseline in the TNFα-inhibitor cohort, and by HAQ disability in the non-biologic cohort. In both cohorts, methotrexate use at baseline was associated with less pain at follow up, whereas glucocortiocosteroids or leflunomide was each associated with SF36-Bodily Pain scores indicating worse pain at 1 year. The overall models were weak (TNFα-inhibitor cohort) or moderate (non-biologic cohort) in explaining pain scores at 1 year (Nagelkerke pseudo-R^2^ = 0.16 and 0.32, respectively).

**Table 2:** Univariate associations with higher than median baseline SF36-Bodily Pain scores in TNFα-inhibitor and non-biologic cohorts

	Tertile or group	TNFα-inhibitor cohort				Non-biologic cohort			
		Original		Imputed (20 sets)	p	Original	p	Imputed (20 sets)	p
Age	Youngest (ref)	1		1		1		1	
	Mid	1.06 (0.97 - 1.17)	0.230	1.06 (0.97 - 1.16)	0.203	1.09 (0.91 - 1.30)	0.366	1.04 (0.89 - 1.22)	0.657
	Oldest	0.98 (0.89 - 1.07)	0.621	0.99 (0.90 - 1.08)	0.815	1.05 (0.88 - 1.26)	0.617	0.97 (0.83 - 1.14)	0.746
Gender	Male (ref)	1		1		1		1	
	Female	**1.11 (1.01 - 1.21)**	**0.033**	**1.10 (1.01 - 1.20)**	**0.033**	1.06 (0.90 - 1.25)	0.504	1.04 (0.90 - 1.20)	0.656
BMI	<25 (ref)	1		1		1		1	
	25 to <30	1.00 (0.91 - 1.10)	0.961	1.01 (0.92 - 1.10)	0.877	1.06 (0.89 - 1.26)	0.534	1.02 (0.88 - 1.19)	0.816
	≥30	**1.23 (1.11 - 1.37)**	**<0.001**	**1.22 (1.11 - 1.34)**	**<0.001**	**1.56 (1.29 - 1.89)**	**<0.001**	**1.44 (1.22 - 1.70)**	**<0.001**
Smoking	Never (ref)	1		1		1		**1**	
	Ex	1.09 (1.00 - 1.19)	0.052	**1.09 (1.00 - 1.18)**	**0.042**	**1.34 (1.14 - 1.58)**	**0.001**	**1.26 (1.08 - 1.46)**	**0.003**
	Current	**1.20 (1.08 - 1.33)**	**<0.001**	**1.19 (1.08 - 1.31)**	**0.001**	**1.58 (1.30 - 1.93)**	**<0.001**	**1.54 (1.30 - 1.83)**	**<0.001**
DAS28	Lowest (ref)	1		1		1		1	
	Mid	**1.49 (1.34 - 1.65)**	**<0.001**	**1.49 (1.35 - 1.65)**	**<0.001**	**2.00 (1.65 - 2.42)**	**<0.001**	**2.03 (1.72 - 2.39)**	**<0.001**
	Highest	**2.94 (2.65 - 3.25)**	**<0.001**	**2.91 (2.64 - 3.21)**	**<0.001**	**4.26 (3.48 - 5.20)**	**<0.001**	**3.99 (3.37 - 4.72)**	**<0.001**
DAS28-P	Lowest (ref)	1		1		1		1	
	Mid	**1.50 (1.36 - 1.66)**	**<0.001**	**1.48 (1.35 - 1.63)**	**<0.001**	**2.03 (1.67 - 2.48)**	**<0.001**	**1.97 (1.67 - 2.33)**	**<0.001**
	Highest	**1.63 (1.47 - 1.80)**	**<0.001**	**1.62 (1.47 - 1.78)**	**<0.001**	**2.45 (2.00 - 2.99)**	**<0.001**	**2.58 (2.17 - 3.05)**	**<0.001**
ESR	Lowest (ref)	1		1		1		1	
	Mid	**1.35 (1.22 - 1.50)**	**<0.001**	**1.34 (1.22 - 1.47)**	**<0.001**	**1.27 (1.05 - 1.53)**	**0.012**	**1.21 (1.03 - 1.42)**	**0.020**
	Highest	**1.85 (1.68 - 2.05)**	**<0.001**	**1.81 (1.65 - 1.99)**	**<0.001**	**1.84 (1.52 - 2.22)**	**<0.001**	**1.69 (1.44 - 1.98)**	**<0.001**
SJC	Lowest (ref)	1		1		1		1	
	Mid	1.09 (0.98 - 1.20)	0.100	1.08 (0.98 - 1.18)	0.106	**1.24 (1.03 - 1.48)**	**0.023**	**1.19 (1.01 - 1.40)**	**0.037**
	Highest	**1.27 (1.16 - 1.40)**	**<0.001**	**1.28 (1.17 - 1.40)**	**<0.001**	**1.50 (1.26 - 1.79)**	**<0.001**	**1.51 (1.29 - 1.76)**	**<0.001**
VAS-GH	Lowest (ref)	1		1		1		1	
	Mid	**1.81 (1.63 - 2.01)**	**<0.001**	**1.83 (1.66 - 2.02)**	**<0.001**	**2.64 (2.19 - 3.18)**	**<0.001**	**2.66 (2.25 - 3.15)**	**<0.001**
	Highest	**5.21 (4.69 - 5.78)**	**<0.001**	**4.89 (4.44 - 5.40)**	**<0.001**	**5.45 (4.48 - 6.63)**	**<0.001**	**5.28 (4.44 - 6.28)**	**<0.001**
TJC	Lowest (ref)	1		1		1		1	
	Mid	**1.35 (1.22 - 1.49)**	**<0.001**	**1.34 (1.22 - 1.47)**	**<0.001**	**1.55 (1.29 - 1.85)**	**<0.001**	**1.63 (1.39 - 1.91)**	**<0.001**
	Highest	**1.91 (1.73 - 2.11)**	**<0.001**	**1.91 (1.74 - 2.10)**	**<0.001**	**3.13 (2.58 - 3.78)**	**<0.001**	**3.01 (2.61 - 3.65)**	**<0.001**
Duration	Shortest (ref)	1		1		1		1	
	Mid	0.98 (0.89 - 1.08)	0.696	0.99 (0.90 - 1.08)	0.799	**1.25 (1.05 - 1.50)**	**0.015**	1.15 (0.98 - 1.35)	0.088
	Longest	1.03 (0.93 - 1.13)	0.612	1.03 (0.95 - 1.13)	0.479	**1.34 (1.12 - 1.60)**	**0.002**	**1.25 (1.07 - 1.47)**	**0.006**
Serology	Seronegative (ref)	1		1		1		1	
	Seropositive	1.08 (0.99 - 1.17)	0.072	1.07 (0.99 - 1.16)	0.071	1.09 (0.94 - 1.26)	0.246	1.04 (0.91 - 1.19)	0.545
Erosions	None (ref)	1		1		1		1	
	At baseline	0.97 (0.91 - 1.06)	0.426	0.97 (0.90 - 1.05)	0.450	1.01 (0.88 - 1.17)	0.882	0.99 (0.87 - 1.13)	0.947
Extra-articular manifestation	No (ref)	1		1		1		1	
	Yes	**1.16 (1.09 - 1.27)**	**<0.001**	**1.16 (1.07 - 1.26)**	**<0.001**	1.16 (0.96 - 1.39)	0.120	1.18 (1.00 - 1.40)	0.053
Co-morbidity	No (ref)	1		1		1		1	
	Yes	**1.30 (1.19 - 1.38)**	**<0.001**	**1.28 (1.19 - 1.38)**	**<0.001**	**1.56 (1.34 - 1.81)**	**<0.001**	**1.52 (1.32 - 1.74)**	**<0.001**
HAQ	Lowest (ref)	1		1		1		1	
	Mid	0.98 (0.89 - 1.08)	0.682	0.99 (0.91 - 1.09)	0.870	**5.04 (4.09 - 6.22)**	**<0.001**	**4.55 (3.80 - 5.44)**	**<0.001**
	Highest	1.10 (0.99 - 1.21)	0.068	1.05 (0.96 - 1.15)	0.329	**19.44 (15.38 - 24.56)**	**<0.001**	**16.30 (13.31 - 19.95)**	**<0.001**
SF36-Vitality	Best (ref)	1		1		1		1	
	Mid	**1.83 (1.64 - 2.05)**	**<0.001**	**1.86 (1.68 - 2.06)**	**<0.001**	**3.19 (2.61 - 3.90)**	**<0.001**	**2.99 (2.51 - 3.56)**	**<0.001**
	Worst	**5.61 (5.05 - 6.22)**	**<0.001**	**5.31 (4.82 - 5.86)**	**<0.001**	**8.89 (7.25 - 10.89)**	**<0.001**	**8.35 (6.97 - 10.00)**	**<0.001**
SF36-Mental Health	Best (ref)	1		1		1		1	
	Mid	**1.92 (1.73 - 2.14)**	**<0.001**	**1.96 (1.78 - 2.17)**	**<0.001**	**2.87 (2.37 - 3.48)**	**<0.001**	**2.65 (2.24 - 3.12)**	**<0.001**
	Worst	**4.39 (3.98 - 4.85)**	**<0.001**	**4.35 (3.96 - 4.78)**	**<0.001**	**7.03 (5.71 - 8.67)**	**<0.001**	**5.65 (4.75 - 6.71)**	**<0.001**
SF36-Physical Function	Best (ref)	1		1		1		1	
	Mid	**2.70 (2.41 - 3.01)**	**<0.001**	**2.55 (2.29 - 2.81)**	**<0.001**	**3.35 (2.75 - 4.09)**	**<0.001**	**3.05 (2.56 - 3.64)**	**<0.001**
	Worst	**9.29 (8.32 - 10.38)**	**<0.001**	**8.17 (7.38 - 9.05)**	**<0.001**	**16.53 (13.13 - 20.81)**	**<0.001**	**12.84 (10.60 - 15.56)**	**<0.001**
Steroids	Not (ref)	1		1		1		**1**	
	At baseline	**1.28 (1.17 - 1.39)**	**<0.001**	**1.25 (1.16 - 1.35)**	**<0.001**	**1.44 (1.21 - 1.71)**	**<0.001**	**1.44 (1.23 - 1.68)**	**<0.001**
Methotrexate	Not (ref)	1		**1**		1		**1**	
	At baseline	**0.77 (0.71 - 0.84)**	**<0.001**	**0.75 (0.69 - 0.82)**	**<0.001**	0.92 (0.79 - 1.07)	0.276	0.92 (0.80 - 1.05)	0.210
Sulphasalazine	Not (ref)	1		**1**		1		**1**	
	At baseline	**0.86 (0.77 - 0.96)**	**0.007**	**0.86 (0.78 - 0.96)**	**0.006**	0.88 (0.75 - 1.03)	0.104	**0.84 (0.73 - 0.96)**	**0.012**
Lefunomide	Not (ref)	1		1		1		1	
	At baseline	1.01 (0.88 - 1.17)	0.882	0.99 (0.86 - 1.14)	0.942	**1.50 (1.20 - 1.87)**	**<0.001**	**1.48 (1.21 - 1.80)**	**<0.001**
Azathioprine	Not (ref)	1		1		1		**1**	
	At baseline	1.03 (0.79 - 1.35)	0.836	1.04 (0.80 - 1.34)	0.796	**1.85 (1.12 - 3.08)**	**0.018**	**1.79 (1.12 - 2.86)**	**0.016**
Hydroxychloroquine	Not (ref)	1		1		1		1	
	At baseline	0.89 (0.78 - 1.02)	0.100	0.90 (0.79 - 1.02)	0.094	0.89 (0.73 - 1.09)	0.280	0.94 (0.78 - 1.13)	0.519
Gold	Not (ref)	1		1		1		1	
	At baseline	1.06 (0.77 - 1.44)	0.750	1.01 (0.75 - 1.37)	0.941	**1.77 (1.24 - 2.52)**	**0.002**	**1.62 (1.18 - 2.21)**	**0.002**

Crude OR (95 % CI) for higher than median SF36-Bodily Pain score at baseline using pooled estimates from the 20 imputed datasets at baseline. The p values have not been adjusted for multiple comparisons. Continuous variables divided into tertiles except for body mass index (BMI). For each variable, the highest tertile is that which signifies worst health (including SF36 variables, which have been reversed). Missing data percentages >5 % for the cases with baseline SF36-Bodily Pain scores were found in the TNF-inhibitor cohort (HAQ(7 %), ESR (7 %), DAS28-ESR (8 %), DAS28-P (8 %), BMI (11 %)) and in the non-biologic cohort (ESR (10 %), DAS28-ESR (11 %), DAS28-P (18 %), BMI (6 %))

*ESR* erythrocyte sedimentation rate, *SJC* swollen joint count, *TJC* tender joint count, *VAS-GH* visual analogue scale-general health. Significant results highlighted in bold

**Table 3:** Univariate predictors of higher than median SF36-Bodily Pain scores after 1 year in TNFα-inhibitor and non-biologic cohorts

	Tertile or group	TNFα-inhibitor cohort				Non-biologic cohort			
		Complete case		Imputed		Complete case		Imputed	
		Crude OR (95 % CI)	p	Crude OR (95 % CI)	p	Crude OR (95 % CI)	p	Crude OR (95 % CI)	p
Age	Youngest (ref)	1		1		1		1	
	Mid	**1.35 (1.21 – 1.51)**	**<0.001**	**1.35 (1.21 – 1.51)**	**<0.001**	**1.39 (1.13 - 1.71)**	**0.002**	**1.39 (1.13 - 1.71)**	**0.002**
	Oldest	**1.67 (1.50 – 1.87)**	**<0.001**	**1.67 (1.50 – 1.87)**	**<0.001**	**1.41 (1.15 - 1.74)**	**0.001**	**1.41 (1.15 - 1.74)**	**0.001**
Gender	Male (ref)	1		1		1		1	
	Female	**0.88 (0.79 – 0.98)**	**0.015**	**0.88 (0.79 – 0.98)**	**0.015**	1.10 (0.91 - 1.33)	0.358	1.10 (0.91 - 1.33)	0.358
BMI	<25 (ref)	1		1		1		1	
	25 to <30	**1.24 (1.11 - 1.38)**	**<0.001**	**1.22 (1.10 - 1.35)**	**<0.001**	1.12 (0.92 - 1.37)	0.264	1.12 (0.92 - 1.35)	0.276
	≥30	**1.53 (1.36 - 1.73)**	**<0.001**	**1.48 (1.33 - 1.66)**	**<0.001**	**1.61 (1.29 - 2.00)**	**<0.001**	**1.60 (1.30 - 1.98)**	**<0.001**
Smoking	Never (ref)	1		1		**1**		**1**	
	Ex	**1.23 (1.11 - 1.36)**	**<0.001**	**1.23 (1.12 - 1.36)**	**<0.001**	**1.33 (1.12 - 1.59)**	**0.002**	**1.37 (1.14 - 1.65)**	**0.001**
	Current	**1.39 (1.23 - 1.56)**	**<0.001**	**1.39 (1.23 - 1.56)**	**<0.001**	1.24 (1.00 - 1.53)	0.054	1.24 (1.00 - 1.53)	0.054
DAS28	Lowest (ref)	1		1		1		1	
	Mid	**1.20 (1.07 – 1.34)**	**0.002**	**1.20 (1.07 – 1.34)**	**0.002**	**1.29 (1.04 - 1.59)**	**0.021**	**1.31 (1.07 - 1.61)**	**0.010**
	Highest	**1.64 (1.47 – 1.84)**	**<0.001**	**1.64 (1.47 – 1.84)**	**<0.001**	**1.96 (1.58 - 2.43)**	**<0.001**	**1.95 (1.59 - 2.39)**	**<0.001**
DAS28-P	Lowest (ref)	1		1		1		1	
	Mid	**1.15 (1.03 – 1.29)**	**0.013**	**1.15 (1.03 – 1.29)**	**0.013**	**1.65 (1.32 - 2.07)**	**<0.001**	**1.59 (1.28 - 1.97)**	**<0.001**
	Highest	**1.46 (1.30 – 1.63)**	**<0.001**	**1.46 (1.30 – 1.63)**	**<0.001**	**2.26 (1.80 - 2.85)**	**<0.001**	**2.20 (1.77 - 2.73)**	**<0.001**
ESR	Lowest (ref)	1		1		1		1	
	Mid	1.00 (0.90 - 1.12)	0.977	1.00 (0.90 - 1.12)	0.978	1.12 (0.91 - 1.38)	0.306	1.06 (0.87 - 1.30)	0.571
	Highest	**1.14 (1.02 – 1.27)**	**0.019**	**1.14 (1.03 - 1.27)**	**0.016**	**1.24 (1.00 - 1.54)**	**0.049**	**1.21 (0.99 - 1.48)**	**0.070**
SJC	Lowest (ref)	1		1		1		1	
	Mid	0.97 (0.87 – 1.08)	0.576	0.97 (0.87 – 1.08)	0.580	0.88 (0.71 - 1.09)	0.268	0.89 (0.73 - 1.10)	0.295
	Highest	1.03 (0.93 - 1.14)	0.608	1.03 (0.93 - 1.14)	0.593	0.96 (0.78 - 1.15)	0.613	0.94 (0.77 - 1.14)	0.549
VAS-GH	Lowest (ref)	1		1		1		1	
	Mid	**1.28 (1.15 – 1.43)**	**<0.001**	**1.27 (1.14 - 1.42)**	**<0.001**	**1.82 (1.48 - 2.24)**	**<0.001**	**1.81 (1.48 - 2.23)**	**<0.001**
	Highest	**1.84 (1.65 - 2.06)**	**<0.001**	**1.84 (1.65 - 2.05)**	**<0.001**	**2.83 (2.29 - 3.49)**	**<0.001**	**2.82 (2.28 - 3.47)**	**<0.001**
TJC	Lowest (ref)	1		1		1		1	
	Mid	**1.12 (1.01 – 1.25)**	**0.040**	**1.12 (1.01 – 1.25)**	**0.037**	**1.54 (1.25 - 1.88)**	**<0.001**	**1.52 (1.25 - 1.86)**	**<0.001**
	Highest	**1.42 (1.27 - 1.59)**	**<0.001**	**1.42 (1.28 - 1.59)**	**<0.001**	**2.11 (1.71 - 2.60)**	**<0.001**	**2.08 (1.69 - 2.56)**	**<0.001**
Duration	Shortest (ref)	1		1		1		1	
	Mid	1.04 (0.93 – 1.16)	0.484	1.04 (0.93 – 1.16)	0.468	**1.44 (1.17 - 1.77)**	**0.001**	**1.44 (1.17 - 1.77)**	**0.001**
	Longest	**1.22 (1.09 – 1.35)**	**<0.001**	**1.21 (1.09 - 1.35)**	**<0.001**	**1.55 (1.27 - 1.91)**	**<0.001**	**1.55 (1.26 - 1.90)**	**<0.001**
Serology	Seronegative (ref)	1		1		1		1	
	Seropositive	0.98 (0.89 - 1.07)	0.657	0.98 (0.89 - 1.07)	0.674	1.05 (0.89 - 1.24)	0.578	1.05 (0.89 - 1.24)	0.608
Erosions	None (ref)	1		1		1		1	
	At baseline	0.92 (0.84 – 1.01)	0.091	0.93 (0.85 - 1.01)	0.096	1.16 (0.98 - 1.37)	0.082	1.16 (0.98 - 1.37)	0.090
Extra-articular manifestation	No (ref)	1		1		1		1	
	Yes	**1.20 (1.09 – 1.32)**	**<0.001**	**1.20 (1.09 – 1.32)**	**<0.001**	1.21 (0.99 - 1.49)	0.071	1.21 (0.99 - 1.49)	0.071
Co-morbidity	No (ref)	**1**		**1**		1		1	
	Yes	**1.66 (1.51 – 1.81)**	**<0.001**	**1.66 (1.51 – 1.81)**	**<0.001**	**1.82 (1.53 - 2.18)**	**<0.001**	**1.82 (1.53 - 2.18)**	**<0.001**
HAQ	Lowest (ref)	1		1		1		1	
	Mid	0.98 (0.87 - 1.09)	0.667	0.98 (0.88 - 1.09)	0.680	**3.03 (2.40 - 3.83)**	**<0.001**	**2.84 (2.28 - 3.54)**	**<0.001**
	Highest	1.01 (0.90 - 1.13)	0.909	1.02 (0.91 - 1.13)	0.804	**10.63 (8.25 - 13.70)**	**<0.001**	**9.17 (7.23 - 11.62)**	**<0.001**
SF36-Vitality	Best (ref)	1		1		1		1	
	Mid	**1.23 (1.09 - 1.38)**	**0.001**	**1.21 (1.08 - 1.35)**	**0.001**	**2.40 (1.90 - 3.03)**	**<0.001**	**2.29 (1.84 - 2.85)**	**<0.001**
	Worst	**1.90 (1.70 - 2.12)**	**<0.001**	**1.84 (1.66 - 2.05)**	**<0.001**	**5.40 (4.30 - 6.80)**	**<0.001**	**4.87 (3.92 - 6.06)**	**<0.001**
SF36-Mental Health	Best (ref)	1		1		1		1	
	Mid	**1.69 (1.51 - 1.90)**	**<0.001**	**1.66 (1.49 - 1.85)**	**<0.001**	**2.08 (1.66 - 2.59)**	**<0.001**	**2.26 (1.84 - 2.78)**	**<0.001**
	Worst	**2.40 (2.15 - 2.68)**	**<0.001**	**2.29 (2.07 - 2.56)**	**<0.001**	**4.26 (3.36 - 5.41)**	**<0.001**	**3.90 (3.15 - 4.83)**	**<0.001**
SF36-Physical Function	Best (ref)	1		1		1		1	
	Mid	**2.01 (1.80 - 2.26)**	**<0.001**	**1.95 (1.74 - 2.17)**	**<0.001**	**3.29 (2.60 0 4.15)**	**<0.001**	**2.70 (2.16 - 3.38)**	**<0.001**
	Worst	**3.83 (3.41 - 4.30)**	**<0.001**	**3.62 (3.23 - 4.04)**	**<0.001**	**9.55 (7.40 - 12.34)**	**<0.001**	**7.72 (6.15 - 9.72)**	**<0.001**
Steroids	Not (ref)	**1**		**1**		**1**		**1**	
	At baseline	**1.58 (1.44 - 1.74)**	**<0.001**	**1.58 (1.44 - 1.74)**	**<0.001**	**1.61 (1.32 - 1.96)**	**<0.001**	**1.61 (1.32 - 1.96)**	**<0.001**
Methotrexate	Not (ref)	1		1		**1**		**1**	
	At baseline	**0.63 (0.57 - 0.69)**	**<0.001**	**0.63 (0.57 - 0.69)**	**<0.001**	**0.79 (0.67 - 0.95)**	**0.010**	**0.79 (0.67 - 0.95)**	**0.010**
Sulphasalazine	Not (ref)	**1**		**1**		1		1	
	At baseline	**0.69 (0.61 - 0.78)**	**<0.001**	**0.69 (0.61 - 0.78)**	**<0.001**	0.85 (0.71 - 1.02)	0.076	0.85 (0.71 - 1.02)	0.076
Lefunomide	Not (ref)	1		1		1		1	
	At baseline	**1.26 (1.07 - 1.49)**	**0.006**	**1.26 (1.07 - 1.49)**	**0.006**	**1.66 (1.28 - 2.16)**	**<0.001**	**1.66 (1.28 - 2.16)**	**<0.001**
Azathioprine	Not (ref)	1		1		**1**		**1**	
	At baseline	**1.44 (1.07 - 1.95)**	**<0.001**	**1.44 (1.07 - 1.95)**	**<0.001**	**2.63 (1.37 - 5.03)**	**0.004**	**2.63 (1.37 - 5.03)**	**0.004**
Hydroxychloroquine	Not (ref)	1		1		**1**		**1**	
	At baseline	**0.67 (0.57 - 0.78)**	**<0.001**	**0.67 (0.57 - 0.78)**	**<0.001**	0.81 (0.64 - 1.03)	0.084	0.81 (0.64 - 1.03)	0.084
Gold	Not (ref)	1		1		1		1	
	At baseline	1.24 (0.87 - 1.76)	0.273	1.24 (0.87 - 1.76)	0.273	**1.50 (1.02 - 2.20)**	**0.047**	**1.50 (1.02 - 2.20)**	**0.047**

Odds ratios (95 % CI) for risk of higher than median pain at 1 year using original data and also pooled estimates of the 20 imputed datasets of baseline variables. The p values have not been adjusted for multiple comparisons. Continuous variables divided into tertiles except for body mass index (BMI). For each variable, the highest tertile is that which signifies worst health (including SF36 variables, which have been reversed). Missing data percentages >5 % for those cases with 1 year pain data available were found in the TNF-inhibitor cohort for HAQ (7 %), ESR (7 %), DAS28-ESR (8 %), DAS28-P (8 %), BMI (11 %), SF36 subscales (5 – 7 %); and in the non-biologic cohort for HAQ (9 %), ESR (10 %), DAS28-ESR (11 %), DAS28-P (19 %), BMI 5 %) and SF36 subscales (8 % - 9 %))

*ESR* erythrocyte sedimentation rate, *SJC* swollen joint count, *TJC* tender joint count, *VAS-GH* visual analogue scale-general health. Significant results highlighted in bold

Logistic regression confirmed that higher DAS28-P, worse mental health, physical function or pain at baseline each independently predicted worse than median SF36-Bodily Pain score indicating worse pain at 1 year in each cohort (Table 4). For example, for DAS28-P at baseline the adjusted odds ratio (95 % CI) for worse pain at 1 year in the TNFα-inhibitor cohort was 1.14 (1.07–1.21), *p* < 0.001 and in the non-biologic control cohort it was 1.27 (1.11 – 1.46), *p* = 0.001. Pain was also predicted by co-morbidities, male gender, higher BMI and current smoking at baseline in the TNFα-inhibitor cohort; and less vitality and lower function in the non-biologic cohort. A sensitivity analysis showed that the presence of variables describing concurrent DMARD and steroid use did not alter the main findings of the models. Two secondary analyses, to further assess whether persistent inflammation would explain the predictors of pain, were performed in the participant subgroups in the TNF-inhibitor cohort who achieved ESR levels <23 for men and <30 for women, or CRP ≤5 g/L respectively after 1 year. A similar logistic regression analysis was performed for each subgroup, adjusting for baseline pain and potential confounding covariates. For the subgroup who achieved a lower ESR at 1 year, predictors of 1 year SF36-Bodily Pain scores indicating worse pain (aOR, (95 % CI)) were worse baseline pain (1.50, (1.29 – 1.76) *p* < 0.001), current smoking (1.45 (1.10 – 1.91, *p* = 0.008), comorbidity (1.37 (1.08 – 1.73), *p* = 0.008), higher DAS28-P (1.17 (1.01 – 1.34) *p* = 0.034), worse mental health (1.36 (1.17 – 1.58) *p* < 0.001), worse physical function (1.80 (1.54 – 2.10) *p* < 0.001), higher BMI (1.16 (1.00 – 1.34) *p* = 0.043) while methotrexate at baseline was negatively associated (0.63 (0.048 – 0.82) *p* = 0.001), each of which was similar to our primary findings. Similarly, for the subgroup who achieved CRP < 5 g/L at 1 year, SF36-Bodily Pain scores indicating worse pain were independently predicted (aOR, 95 % CI) by baseline current smoking (1.97, 1.26 – 3.07, *p* = 0.003) and higher DAS28-P (1.26, 1.02 – 1.56, *p* = 0.030).

**Table 4:** Multivariable-adjusted logistic regression for predictors of higher than median SF36-Bodily Pain scores after 1 year in TNFα-inhibitor and non-biologic cohorts

		Higher than median pain score - 1 year							
Variable at baseline	Tertile or group at baseline	TNFα-inhibitors cohort				Non-biologic cohort			
		Complete case		Imputed		Complete case		Imputed	
		aOR (95 % CI)	p	aOR (95 % CI)	p	aOR (95 % CI)	p	aOR (95 % CI)	p
Age	Tertiles	**1.14 (1.06 - 1.22)**	**0.001**	**1.19 (1.12 - 1.27)**	**<0.001**	1.06 (0.90 - 1.25)	0.470	1.03 (0.89 - 1.18)	0.728
Gender	Female	**0.84 (0.73 - 0.96)**	**0.013**	**0.84 (0.74 - 0.94)**	**0.003**	0.86 (0.65 - 1.14)	0.296	0.90 (0.71 - 1.15)	0.317
BMI	WHO groups	**1.12 (1.05 - 1.21)**	**0.002**	**1.13 (1.05 - 1.21)**	**0.001**	1.06 (0.91 - 1.24)	0.463	1.07 (0.93 - 1.22)	0.353
Smoking	Current	**1.25 (1.07 - 1.46)**	**0.006**	**1.28 (1.11 - 1.47)**	**<0.001**	0.98 (0.70 - 1.38)	0.924	1.16 (0.87 - 1.54)	0.317
Smoking	Ex-	1.03 (0.90 - 1.17)	0.678	1.05 (0.94 - 1.17)	0.421	1.14 (0.87 - 1.49)	0.344	1.16 (0.92 - 1.46)	0.698
DAS28	Tertiles	1.02 (0.95 - 1.10)	0.638	1.01 (0.95 - 1.08)	0.640	**0.80 (0.68 - 0.94)**	**0.007**	**0.86 (0.75 - 0.99)**	**0.041**
DAS28-P	Tertiles	**1.13 (1.05 - 1.21)**	**0.001**	**1.14 (1.07 - 1.21)**	**<0.001**	**1.26 (1.08 - 1.47)**	**0.003**	**1.27 (1.11 - 1.46)**	**0.001**
Duration	Tertiles	1.04 (0.97 - 1.12)	0.264	1.01 (0.95 - 1.08)	0.660	1.08 (0.92 - 1.27)	0.326	1.08 (0.94 - 1.24)	0.265
Serology	Positive	0.91 (0.81 - 1.03)	0.127	0.96 (0.86 - 1.07)	0.440	1.01 (0.79 - 1.30)	0.919	1.04 (0.84 - 1.29)	0.696
Erosions	Positive	0.93 (0.82 - 1.05)	0.244	0.94 (0.85 - 1.05)	0.282	0.98 (0.75 - 1.27)	0.848	1.02 (0.82 - 1.28)	0.838
Extra-articular manifestation	Yes	1.09 (0.96 - 1.23)	0.191	1.06 (0.95 - 1.18)	0.305	1.03 (0.77 - 1.39)	0.840	0.95 (0.74 - 1.22)	0.698
Co-morbidity	Yes	**1.27 (1.12 - 1.43)**	**<0.001**	**1.29 (1.16 - 1.43)**	**<0.001**	**1.31 (1.01 - 1.70)**	**0.041**	1.24 (1.00 - 1.55)	0.055
HAQ	Tertiles	0.98 (0.91 - 1.05)	0.553	0.99 (0.93 - 1.05)	0.703	**1.44 (1.17 - 1.78)**	**<0.001**	**1.55 (1.29 - 1.87)**	**<0.001**
SF36-Vitality	Tertiles	0.95 (0.88 1.03)	0.183	0.97 (0.91 - 1.04)	0.382	**1.31 (1.10 - 1.55)**	**0.002**	**1.21 (1.04 - 1.40)**	**0.015**
SF36-Mental Health	Tertiles	**1.25 (1.17 - 1.35)**	**<0.001**	**1.23 (1.15 - 1.31)**	**<0.001**	**1.29 (1.08 - 1.53)**	**0.005**	**1.29 (1.11 - 1.50)**	**0.001**
SF36-Physical Function	Tertiles	**1.51 (1.40 - 1.63)**	**<0.001**	**1.47 (1.37 - 1.57)**	**<0.001**	**1.44 (1.18 - 1.76)**	**<0.001**	**1.36 (1.14 - 1.62)**	**<0.001**
SF36-Bodily Pain	Tertiles	**1.49 (1.38 - 1.62)**	**<0.001**	**1.46 (1.36 - 1.57)**	**<0.001**	**1.99 (1.64 - 2.42)**	**<0.001**	**1.77 (1.49 - 2.10)**	**<0.001**
Steroid	At baseline	**1.24 (1.09 - 1.40)**	**0.001**	**1.28 (1.15 - 1.43)**	**<0.001**	1.25 (0.94 - 1.67)	0.124	**1.35 (1.06 - 1.73)**	**0.016**
Methotrexate	At baseline	**0.80 (0.71 - 0.91)**	**0.001**	**0.81 (0.72 - 0.92)**	**<0.001**	0.84 (0.61 - 1.14)	0.250	0.91 (0.70 - 1.19)	0.508
Sulphasalazine	At baseline	**0.80 (0.68 - 0.94)**	**0.008**	**0.81 (0.70 - 0.93)**	**0.003**	0.87 (0.65 - 1.16)	0.337	0.98 (0.76 - 1.25)	0.845
Lefunomide	At baseline	1.18 (0.97 - 1.44)	0.107	**1.20 (1.01 - 1.43)**	**0.038**	1.13 (0.73 - 1.74)	0.595	1.18 (0.81 - 1.71)	0.394
Azathioprine	At baseline	**1.40 (1.02 - 1.93)**	**0.036**	1.18 (0.89 - 1.56)	0.245	1.26 (0.49 - 3.24)	0.637	1.87 (0.83 - 4.19)	0.131
Hydroxychloroquine	At baseline	0.83 (0.68 - 1.01)	0.061	**0.83 (0.70 - 0.99)**	**0.034**	0.82 (0.59 - 1.15)	0.248	0.90 (0.68 - 1.20)	0.473
Gold	At baseline	0.93 (0.65 - 1.31)	0.664	1.03 (0.75 - 1.40)	0.858	1.20 (0.69 - 2.07)	0.523	1.22 (0.75 - 1.99)	0.428

Two multi-variable adjusted logistic regression models for higher than median pain score at 1 year follow up using data from the 20 imputed datasets of baseline variables. Continuous variables divided into tertiles except for body mass index (BMI). For each variable, the highest tertile is that which signifies worst health (including SF36 variables, which have been reversed). Significant results are highlighted in bold

Discontinuation of the initial TNFα-inhibitor was reported by 32 % (3475/10949) at 1 year. Of patients with median SF36-Bodily Pain scores indicating worse pain at 1 year, 39 % (1302/3376) had discontinued their original TNF inhibitor, as compared to 17 % (722/4140) of those with less than median pain. Mean (s.d.) SF36-Bodily Pain scores at 1 year were worse in those who stopped or changed their TNFα-inhibitors (29 (9)) than in those who continued (35 (10), *p* < 0.001). SF36-Bodily Pain scores at 1 year were worse both in those who discontinued TNFα-inhibitors due to inefficacy (28 (9)) and in those who discontinued due to adverse events (29 (9)), compared with those who continued (each *p* < 0.05). Univariate predictors of discontinuation included higher baseline DAS28, extra-articular disease, co-morbidities, worse physical function, mental health, vitality and Bodily Pain scores (Additional file 1: Table S2). Logistic regression showed independent prediction of discontinuation by baseline smoking, extra-articular disease, comorbidities, worse physical function and Bodily Pain score at baseline, and baseline non-biologic treatment (Table 5). People with a baseline treatment that did not include methotrexate or sulphasalazine were more likely to discontinue TNFα-inhibitors, whether due to inefficacy or to adverse events. Shorter disease duration and higher pain at baseline were stronger predictors of TNFα-inhibitor discontinuation due to inefficacy rather than to adverse events, whereas age, poorer physical function, longer disease duration and extra-articular disease were stronger predictors of discontinuation due to adverse events rather than inefficacy (Table 5). The main model showed a weak effect for predicting discontinuation for any cause (inefficacy or adverse events) (Hosmer-Lemeshow *χ*^2^ = 3.1, *p* = 0.925; and the Nagelkerke pseudo-R^2^ = 0.04).

**Table 5:** Logistic regression for discontinuation of TNFα-inhibitors within 1 year of their initiation

		Discontinuation of TNFα-inhibitors											
Variable at baseline	Tertile or group at baseline	All reasons				Lack of Efficacy				Adverse Drug Reaction			
		Complete case		Imputed		Complete case		Imputed		Complete case		Imputed	
		aOR (95 % CI)	p	aOR (95 % CI)	p	aOR (95 % CI)	p	aOR (95 % CI)	p	aOR (95 % CI)	p	aOR (95 % CI)	p
Age	Tertiles	1.03 (0.96 - 1.10)	0.384	1.04 (0.98 - 1.11)	0.155	0.99 (0.90 - 1.09)	0.856	1.02 (0.94 - 1.11)	0.643	1.07 (0.98 - 1.17)	0.149	**1.10 (1.02 - 1.19)**	**0.020**
Gender	Female	1.12 (0.99 - 1.27)	0.062	**1.16 (1.04 - 1.28)**	**0.007**	1.18 (0.99 - 1.40)	0.063	**1.18 (1.02 - 1.37)**	**0.029**	1.11 (0.94 - 1.32)	0.212	**1.14 (0.99 - 1.32)**	**0.077**
BMI	WHO	0.99 (0.93 - 1.05)	0.673	0.98 (0.93 - 1.04)	0.538	1.03 (0.94 - 1.12)	0.540	1.01 (0.93 - 1.09)	0.826	0.94 (0.86 - 1.03)	0.173	0.94 (0.87 - 1.02)	0.141
Smoking	Current	**1.24 (1.09 - 1.42)**	**0.002**	**1.23 (1.09 - 1.38)**	**0.001**	**1.25 (1.04 - 1.50)**	**0.017**	**1.21 (1.03 - 1.43)**	**0.019**	**1.23 (1.02 - 1.47)**	**0.031**	**1.19 (1.02 - 1.40)**	**0.033**
Smoking	Ex	1.07 (0.96 - 1.21)	0.237	1.08 (0.98 - 1.20)	0.101	1.02 (0.87 - 1.21)	0.791	1.05 (0.91 - 1.21)	0.489	1.12 (0.96 - 1.32)	0.151	1.13 (0.99 - 1.30)	0.081
DAS28	Tertiles	1.04 (0.98 - 1.11)	0.226	1.02 (0.96 - 1.07)	0.580	1.08 (0.99 - 1.19)	0.087	1.04 (0.97 - 1.13)	0.285	1.02 (0.93 - 1.11)	0.711	0.99 (0.91 - 1.07)	0.738
DAS28-P	Tertiles	0.99 (0.93 - 1.05)	0.719	0.98 (0.93 - 1.04)	0.567	0.96 (0.88 - 1.05)	0.410	0.98 (0.91 - 1.06)	0.549	0.99 (0.91 - 1.08)	0.876	1.00 (0.93 - 1.08)	0.993
Duration	Tertiles	0.98 (0.92 - 1.05)	0.626	1.01 (0.95 - 1.06)	0.841	**0.87 (0.79 - 0.95)**	**0.002**	**0.89 (0.82 - 0.96)**	**0.004**	1.09 (1.00 - 1.19)	0.053	**1.09 (1.01 - 1.17)**	**0.037**
Serology	Positive	1.04 (0.93 - 1.16)	0.523	1.01 (0.92 - 1.11)	0.830	0.94 (0.81 - 1.09)	0.432	0.92 (0.81 - 1.05)	0.213	1.09 (0.93 - 1.26)	0.283	1.07 (0.94 - 1.21)	0.344
Erosions	Positive	0.93 (0.84 - 1.04)	0.224	0.93 (0.85 - 1.03)	0.151	0.96 (0.83 - 1.12)	0.619	0.95 (0.84 - 1.09)	0.482	0.93 (0.80 - 1.09)	0.368	0.94 (0.82 - 1.07)	0.324
Extra-articular manifestation	Yes	**1.17 (1.05 - 1.31)**	**0.004**	**1.17 (1.07 - 1.29)**	**0.001**	1.08 (0.93 - 1.27)	0.302	1.06 (0.93 - 1.22)	0.386	**1.33 (1.15 - 1.53)**	**<0.001**	**1.32 (1.16 - 1.50)**	**<0.001**
Co-morbidity	Yes	1.11 (1.00 - 1.24)	0.059	**1.14 (1.04 - 1.25)**	**0.005**	1.12 (0.97 - 1.31)	0.129	**1.15 (1.01 - 1.31)**	**0.040**	1.11 (0.95 - 1.28)	0.182	1.13 (1.00 - 1.28)	0.058
HAQ	Tertiles	0.95 (0.89 - 1.01)	0.097	0.97 (0.92 - 1.02)	0.277	0.94 (0.86 - 1.02)	0.143	0.95 (0.88 - 1.03)	0.185	0.94 (0.86 - 1.02)	0.138	0.97 (0.90 - 1.05)	0.444
SF36-Vitality	Tertiles	0.99 (0.92 - 1.06)	0.771	0.98 (0.92 - 1.04)	0.471	1.06 (0.96 - 1.17)	0.248	1.05 (0.96 - 1.14)	0.306	0.96 (0.87 - 1.06)	0.402	0.96 (0.89 - 1.04)	0.304
SF36-Mental Health	Tertiles	1.02 (0.96 - 1.10)	0.531	1.02 (0.96 - 1.08)	0.540	1.00 (0.91 - 1.09)	0.923	1.00 (0.91 - 1.08)	0.900	1.02 (0.94 - 1.12)	0.608	1.02 (0.94 - 1.11)	0.587
SF36-Physical Function	Tertiles	**1.14 (1.06 - 1.22)**	**<0.001**	**1.15 (1.08 - 1.22)**	**<0.001**	1.07 (0.97 - 1.18)	0.171	1.08 (0.99 - 1.18)	0.082	**1.25 (1.14 - 1.38)**	**<0.001**	**1.24 (1.14 - 1.35)**	**<0.001**
SF36-Bodily Pain	Tertiles	**1.13 (1.05 - 1.22)**	**0.002**	**1.12 (1.05 - 1.20)**	**<0.001**	**1.23 (1.10 - 1.36)**	**<0.001**	**1.22 (1.11 - 1.33)**	**<0.001**	0.99 (0.90 - 1.10)	0.885	1.04 (0.95 - 1.14)	0.389
Steroid	At baseline	0.99 (0.88 - 1.11)	0.796	0.96 (0.87 - 1.06)	0.436	0.98 (0.83 - 1.15)	0.777	0.93 (0.80 - 1.07)	0.288	0.96 (0.81 - 1.13)	0.613	0.94 (0.82 - 1.09)	0.380
Methotrexate	At baseline	**0.69 (0.61 - 0.78)**	**<0.001**	**0.70 (0.63 - 0.78)**	**<0.001**	**0.73 (0.61 - 0.87)**	**<0.001**	**0.70 (0.60 - 0.82)**	**<0.001**	**0.60 (0.50 - 0.71)**	**<0.001**	**0.58 (0.50 - 0.67)**	**<0.001**
Sulphasalazine	At baseline	**0.74 (0.63 - 0.86)**	**<0.001**	**0.74 (0.65 - 0.84)**	**<0.001**	0.85 (0.69 - 1.04)	0.111	**0.81 (0.68 - 0.97)**	**0.024**	**0.61 (0.49 - 0.77)**	**<0.001**	**0.66 (0.55 - 0.80)**	**<0.001**
Lefunomide	At baseline	1.07 (0.88 - 1.30)	0.524	1.01 (0.85 - 1.19)	0.928	**1.36 (1.05 - 1.75)**	**0.019**	1.19 (0.95 - 1.49)	0.133	0.86 (0.65 - 1.13)	0.267	0.88 (0.69 - 1.11)	0.262
Hydroxychloroquine	At baseline	**0.82 (0.68 - 0.98)**	**0.032**	**0.85 (0.73 - 1.00)**	**0.046**	0.87 (0.67 - 1.12)	0.273	0.90 (0.73 - 1.13)	0.364	0.79 (0.61 - 1.04)	0.134	0.88 (0.71 - 1.10)	0.274
Gold	At baseline	**0.62 (0.41 - 0.95)**	**0.026**	0.70 (0.48 - 1.01)	0.055	0.60 (0.32 - 1.10)	0.097	0.68 (0.40 - 1.16)	0.161	0.65 (0.37 - 1.14)	0.613	0.66 (0.40 - 1.11)	0.115

Three multi-variable adjusted logistic regression models for TNFα-inhibitor discontinuation at 1 year using data from 20 imputed datasets at baseline (table contains all variables included in the models). Continuous variables divided into tertiles except for body mass index (BMI). For each variable, the highest tertile is that which signifies worst health (including SF36 variables, which have been reversed). Significant results are highlighted in bold

## Discussion

In this study we found that pain persists in many people with RA, despite the initial clinically significant [34] improvement upon commencing a TNFα-inhibitor. Several baseline factors, including a high contribution of patient reported factors to disease activity scores (high DAS28-P), poor mental health, poor physical function and the presence of co-morbidities, predicted pain outcomes after either TNFα-inhibitors or traditional DMARDs. Discontinuation of TNFα-inhibitors within 1 year from their initiation was associated with persistent pain, and predicted by baseline factors that overlapped with predictors of 1 year pain outcomes, including smoking, and higher baseline pain.

We identified a broad range of demographic and clinical factors that predicted 1 year pain outcomes in this study. However, teasing apart inflammatory and non-inflammatory pain mechanisms in the clinic can be extremely difficult. Although all 4 components of DAS28 are related to inflammation, the TJC and VAS-GH have also been associated with non-inflammatory pain mechanisms and concurrent fibromyalgia [10]. Derived indices such as DAS28-P might provide insight into the balance of different pain mechanisms. In a previous study of people with early RA commencing non-biologic DMARDs we found that higher DAS28-P scores predicted less improvement in pain at 12 months [9]. Early RA differs from established disease in several important aspects, such as the establishment of chronic inflammation and psychological adaptions to chronic pain, plus the effects of new DMARD treatments on DMARD-naïve patients. The current analysis of data from the BSRBR confirms and extends to established disease our findings in early RA, showing that high baseline DAS28-P was also associated with worse pain after commencing TNFα-inhibitors. High DAS28-P might be an indicator that pain is driven by non-inflammatory mechanisms such as central pain processing, resulting in disproportionately high tender joint counts and VAS-GH [10, 14, 15]. DAS28-P might be higher in people with fibromyalgia, either with [15] or without [14] RA, and was associated with widespread sensitivity to pressure induced pain, even at sites distant to inflamed joints in people with longstanding RA and DAS28 > 3.1 [10]. These observations are consistent with DAS28-P representing an index of augmented pain processing in RA, which might have utility for epidemiology studies where more detailed pain measurements cannot be obtained, and in retrospective studies or secondary data analysis. Targeting inflammatory pain mechanisms alone, either with TNFα-inhibitors or traditional DMARDs, may be insufficient to adequately manage pain in these patients.

Other baseline factors that consistently predicted 1 year pain outcomes were poor mental health, poor physical function and the presence of co-morbidities. Poor mental health predicts worse pain prognosis in a range of chronic conditions [35]. Fear and low mood represent emotional components of the pain experience, and might impair uptake or response to treatment [36]. Furthermore, psychological factors might specifically interact with central pain processing to augment or maintain chronic pain [5]. Physiotherapy and some exercise regimes can improve outcomes [37, 38], and our data raise the possibility that poor physical function may be a barrier to responding to treatments. Prediction of poor pain outcomes by baseline physical function was observed in both cohorts, but was particularly strong in the non-biologic DMARD cohort, and when physical function was measured by SF36 rather than HAQ. Differences between data from the 2 questionnaires might reflect their different emphases on mobility (SF36) or hand function (HAQ). We found that the presence of comorbidities predicted poorer pain prognosis. Osteoarthritis is a possible additional source of pain in those with RA and might be prevalent in the aged population. Further work could investigate whether comorbidities are a source of pain, or have been barriers to effective RA treatment. Poor mental health, physical function and comorbidities might each be amenable to treatment in their own right, but randomised clinical trials would be required to test whether targeting them can improve pain. Gender was found to predict future pain in our previous study of early RA using similar methodologies to this one [9]. This association may be due to reporting preferences, different pain sensitivities or different manifestations of RA across the genders. We found no association between baseline erosions and future pain but more detailed assessment would be required to determine whether pain in RA might be partly mediated by progressive joint damage.

We have found that factors that predicted early discontinuation of TNFα-inhibitors only partially overlapped with those that predicted poor pain outcomes. Pain levels were higher at baseline and at 1 year in those who discontinued TNFα-inhibitors, suggesting that inadequate pain control contributes to some of the decisions involved in discontinuation. We were not able to exclude in this study the possibility that worse pain outcomes might be caused by TNFα-inhibitor discontinuation, and pain might further improve if more effective biologic therapy were initiated. However, our secondary analysis on those with low CRP levels and those in remission suggests that persistent inflammation was not the sole reason for worse pain during follow up. The decision to discontinue medication is based upon the balance between efficacy and adverse events, and so discontinuation is often difficult to exclusively assign to one or other reason. However, in the current study, pain predicted discontinuation due to inefficacy, and not due to adverse events. We cannot be certain from our study of any causal relationship between pain and TNFα-inhibitor discontinuation, and further research would be required to determine whether more effective analgesic strategies would facilitate continuation of concurrently administered TNFα-inhibitors.

Several factors limit interpretation of our findings. We have identified baseline factors that predict 1 year pain outcomes in people with RA, but further research is required to determine what interventions can improve on these outcomes. Chanelling bias might have contributed to observed associations between baseline non-DMARD biologic medication use and 1 year pain, such that medications should not be interpreted as representing independent risk factors. Exploration of the effects of treatments upon RA pain is best addressed through randomised controlled trials, although it was important to include DMARD treatment in our models to ensure that they did not confound our results. Patient choice to use analgesics is moderated by a wide range of factors in addition to pain severity, including efficacy, adverse events and beliefs about medications and illness [39]. Further research would be required, therefore, to determine whether factors found in the current study to predict pain also predict healthcare utilisation due to that pain. Our models predicting 1 year pain were based upon an outcome variable (SF36 Bodily Pain score) with a moderate percentage of missingness due to attrition. The use of multiple imputation reduced the total level of missingness in this study, but might have selected from subsets of our data showing some degree of bias. Our models had only weak to moderate effects in explaining pain outcomes, and unmeasured factors might make important contributions to predicting Bodily Pain scores. In particular, measurements of centrally-augmented pain, or fibromyalgia classification criteria or a pain Manikin, would also have been useful to verify our findings. DAS28-P was associated with fibromyalgia classification in a study of 50 people with RA [10], but data were not available to determine fibromyalgia status in the BSRBR cohorts. Even though the BSRBR cohorts are large, un-measured factors, such as gene polymorphisms or additional treatment details, could have mediated or obscured associations between baseline characteristics and pain outcomes. The search for additional risk factors for worse pain or predictive markers requires further work. Similarities between our findings in the BSRBR and early RA [9] cohorts suggest that they might be generalisable to some other RA populations. Participants in the TNFα-inhibitor cohort are likely to be representative of patients commenced on their first biologic agent in the UK, but caution should be exercised before generalising our findings. Further work should determine whether the factors that we identified predict longer term outcomes. Our findings suggest possible mechanisms of pain but this study is not sufficient to justify stratification of treatment based on these factors alone.

## Conclusions

In conclusion, pain persists in many people with treated RA, even when inflammation responds to treatment. Worse 1 year pain outcomes are predicted by factors different to those typically found to predict inflammatory disease activity, and include baseline Bodily Pain scores, the proportion of DAS28 attributable to patient-reported components, worse physical function or mental health, and co-morbidities. Worse pain at baseline also predicts TNFα-inhibitor discontinuation within 1 year. Improved pain management should complement inflammatory disease suppression in RA.

## Abbreviations

Aza, azathioprine; BSRBR, British Society for Rheumatology Biologics Register; DAS28, 28 joint disease activity score; ESR, erythrocyte sedimentation rate; EULAR, European League Against Rheumatism; HCQ, hydroxychloroquine; Lef, leflunomide; MTX, methotrexate; QoL, quality of life; SF36, short form-36 questionnaire; SJC, swollen joint count; SSZ, sulphasalazine; TJC, tender joint count; VAS, visual analogue scale

## Additional file

Additional file 1: Univariate associations with discontinuation of TNFα-inhibitor. (DOCX 54 kb)
