# Supplementary material for: Factors predicting pain and early discontinuation of tumour necrosis factor-α-inhibitors in people with rheumatoid arthritis: results from the British society for rheumatology biologics register
Source: BMC Musculoskelet Disord. 2016 Aug 12;17:337. doi: 10.1186/s12891-016-1192-7 (PMC4982340; doi:10.1186/s12891-016-1192-7)
Supplement: Additional file 1: — Univariate associations with discontinuation of TNFα-inhibitor. (DOCX 54 kb) [file 12891_2016_1192_MOESM1_ESM.docx]

**APPENDICES/SUPPLEMENTS**

**Supplementary Table 1: Description of variables for both cohorts**

|  |  | TNFα-inhibitor cohort | | | | | |  | Non-biologic cohort | | | | | |
| --- | --- | --- | --- | --- | --- | --- | --- | --- | --- | --- | --- | --- | --- | --- |
|  |  | Original | | |  | Imputation#1 | |  | Original | | |  | Imputation#1 | |
| Tertile or group | | Range | n= | Present% |  | Range | n= |  | Range | n= | Present% |  | Range | n= |
| Age | Youngest (ref) | 16-51 | 3890 | 100% |  | 16-51 | 3890 |  | 18-56 | 1254 | 100% |  | 18-56 | 1254 |
|  | Mid | 52-62 | 4264 |  |  | 52-62 | 4264 |  | 57-66 | 1194 |  |  | 57-66 | 1194 |
|  | Oldest | 63-89 | 3841 |  |  | 63-89 | 3841 |  | 67-91 | 1184 |  |  | 67-91 | 1184 |
| Gender | Male (ref) |  | 2863 | 100% |  |  | 2863 |  |  | 998 | 100% |  |  | 998 |
|  | Female |  | 9132 |  |  |  | 9132 |  |  | 2634 |  |  |  | 2634 |
| BMI | <25 (ref) | 15-25 | 4485 | 88% |  | 15-25 | 4485 |  | 15-25 | 1324 | 94% |  | 15-25 | 1400 |
|  | 25 to <30 | 25-30 | 3403 |  |  | 25-30 | 3403 |  | 25-30 | 1205 |  |  | 25-30 | 1267 |
|  | ≥30 | 30-100 | 2614 |  |  | 30-100 | 2614 |  | 30-100 | 891 |  |  | 30-100 | 960 |
| Smoking | Never (ref) |  | 4785 | 99% |  |  | 4828 |  |  | 1318 | 100% |  |  | 1326 |
|  | Ex |  | 4496 |  |  |  | 4524 |  |  | 1438 |  |  |  | 1444 |
|  | Current |  | 2626 |  |  |  | 2643 |  |  | 858 |  |  |  | 862 |
| DAS28 | Lowest (ref) | 1.2-6.1 | 3641 | 91% |  | 1.2-6.1 | 3641 |  | 0.3-4.6 | 1071 | 88% |  | 0.3-4.6 | 1210 |
|  | Mid | 6.1-7.0 | 3642 |  |  | 6.1-7.0 | 3642 |  | 4.6-5.7 | 1072 |  |  | 4.6-5.7 | 1211 |
|  | Highest | 7.0-9.4 | 3641 |  |  | 7.0-9.4 | 3641 |  | 5.7-8.9 | 1071 |  |  | 5.7-8.9 | 1211 |
| DAS28-P | Lowest (ref) | 0.08-0.45 | 3626 | 91% |  | 0.08-0.45 | 3626 |  | 0.00-0.41 | 988 | 82% |  | 0.00-0.41 | 1118 |
|  | Mid | 0.45-0.51 | 3626 |  |  | 0.45-0.51 | 3626 |  | 0.41-0.48 | 989 |  |  | 0.41-0.48 | 1119 |
|  | Highest | 0.51-0.88 | 3626 |  |  | 0.51-0.88 | 3626 |  | 0.48-0.85 | 988 |  |  | 0.48-0.85 | 1118 |
| ESR | Lowest (ref) | 1-29 | 3735 | 92% |  | 1-30 | 3979 |  | 1-20 | 1095 | 89% |  | 1-21 | 1193 |
|  | Mid | 30-55 | 3655 |  |  | 30-56 | 3998 |  | 21-40 | 1092 |  |  | 21-41 | 1219 |
|  | Highest | 56-215 | 3645 |  |  | 56-215 | 4018 |  | 41-138 | 1059 |  |  | 41-138 | 1220 |
| SJC | Lowest (ref) | 0-8 | 4179 | 97% |  | 0-8 | 4315 |  | 0-3 | 1340 | 98% |  | 0-3 | 1355 |
|  | Mid | 9-13 | 3471 |  |  | 9-13 | 3590 |  | 4-6 | 988 |  |  | 4-6 | 1006 |
|  | Highest | 14-28 | 3953 |  |  | 14-28 | 4090 |  | 7-28 | 1232 |  |  | 7-28 | 1271 |
| VAS-GH | Lowest (ref) | 0-69 | 3880 | 96% |  | 0-68 | 3998 |  | 0-48 | 1180 | 98% |  | 0-48 | 1209 |
|  | Mid | 70-81 | 3829 |  |  | 68-81 | 4045 |  | 49-69 | 1174 |  |  | 49-69 | 1198 |
|  | Highest | 82-100 | 3841 |  |  | 81-100 | 3952 |  | 70-100 | 1215 |  |  | 70-100 | 1225 |
| TJC | Lowest (ref) | 0-11 | 3735 | 97% |  | 0-11 | 3855 |  | 0-4 | 1186 | 98% |  | 0-4 | 1203 |
|  | Mid | 12-19 | 4040 |  |  | 12-19 | 4207 |  | 5-10 | 1270 |  |  | 5-10 | 1288 |
|  | Highest | 20-28 | 3832 |  |  | 20-28 | 3933 |  | 11-28 | 1107 |  |  | 11-28 | 1141 |
| Duration | Shortest (ref) | 0-7 | 4013 | 99% |  | 0-7 | 4028 |  | 0-2 | 1196 | 99% |  | 0-2 | 1198 |
|  | Mid | 8-15 | 3880 |  |  | 8-15 | 3909 |  | 3-11 | 1220 |  |  | 3-11 | 1230 |
|  | Longest | 16-72 | 4007 |  |  | 16-72 | 4058 |  | 12-65 | 1193 |  |  | 12-65 | 1204 |
| Serology | Seronegative (ref) |  | 4229 | 100% |  |  | 4229 |  |  | 1526 | 100% |  |  | 1526 |
|  | Seropositive |  | 7757 |  |  |  | 7766 |  |  | 2103 |  |  |  | 2106 |
| Erosions | None (ref) |  | 4509 | 100% |  |  | 4515 |  |  | 1952 | 100% |  |  | 1953 |
|  | At baseline |  | 7464 |  |  |  | 7480 |  |  | 1678 |  |  |  | 1679 |
| Extra-articular manifestation | No (ref) |  | 8519 | 100% |  |  | 8519 |  |  | 2967 | 100% |  |  | 2967 |
|  | Yes |  | 3476 |  |  |  | 3476 |  |  | 665 |  |  |  | 665 |
| Co-morbidity | No (ref) |  | 4799 | 100% |  |  | 4799 |  |  | 1286 | 100% |  |  | 1286 |
|  | Yes |  | 7196 |  |  |  | 7196 |  |  | 2346 |  |  |  | 2346 |
| HAQ | Lowest (ref) | 0-1.9 | 3383 | 93% |  | 0-1.9 | 3762 |  | 0.0-1.1 | 981 | 80% |  | 0.0-1.2 | 1210 |
|  | Mid | 1.9-2.4 | 3817 |  |  | 1.9-2.4 | 4106 |  | 1.3-1.9 | 998 |  |  | 1.2-1.9 | 1285 |
|  | Highest | 2.4-3.0 | 3921 |  |  | 2.4-3.0 | 4127 |  | 2.0-3.0 | 935 |  |  | 1.9-3.0 | 1137 |
| SF36-Vitality | Best (ref) | 37-71 | 3473 | 90% |  | 36-71 | 3948 |  | 46-71 | 940 | 80% |  | 43-71 | 1188 |
|  | Mid | 29-36 | 3270 |  |  | 27-36 | 3682 |  | 36-43 | 896 |  |  | 33-43 | 1151 |
|  | Worst | 20-27 | 4082 |  |  | 5-27 | 4365 |  | 20-33 | 1075 |  |  | 8-33 | 1293 |
| SF36-Mental Health | Best (ref) | 46-65 | 3868 | 90% |  | 46-82 | 4199 |  | 54-65 | 877 | 80% |  | 52-73 | 1290 |
|  | Mid | 38-45 | 3085 |  |  | 35-46 | 3566 |  | 41-52 | 1122 |  |  | 41-52 | 1156 |
|  | Worst | 10-35 | 3858 |  |  | 4-35 | 4230 |  | 10-38 | 900 |  |  | 6-41 | 1186 |
| SF36-Physical Function | Best (ref) | 18-56 | 3836 | 91% |  | 18-56 | 4301 |  | 31-56 | 946 | 80% |  | 28-65 | 1210 |
|  | Mid | 10-17 | 3455 |  |  | 10-17 | 3818 |  | 15-28 | 1065 |  |  | 15-28 | 1121 |
|  | Worst | 5-9 | 3615 |  |  | 0-10 | 3876 |  | 5-15 | 903 |  |  | 0-15 | 1301 |
| SF36-Bodily Pain | Best (ref) | 26-59 | 3258 | 91% |  | 25-59 | 3782 |  | 34-59 | 757 | 80% |  | 34-59 | 1027 |
|  | Mid | 24-25 | 3668 |  |  | 21-25 | 3919 |  | 26-34 | 1098 |  |  | 25-34 | 1356 |
|  | Worst | 16-21 | 3991 |  |  | 0-21 | 4294 |  | 16-25 | 1061 |  |  | 6-25 | 1249 |

**Each variable’s original data and example imputed data ranges and tertiles/groups are shown. The first imputation dataset (out of 20) was used for example.**

**Present% shows the percentage of the original data that were not missing at baseline.**

**Supplementary Table 2: Univariate predictors of discontinuation of TNFα-inhibitors from the Biologics cohort**

|  | Tertile or group | Discontinuation of TNFα-inhibitors | | | |  | Discontinuation due to Inefficacy | | | |  | Discontinuation due to Adverse Event | | | |
| --- | --- | --- | --- | --- | --- | --- | --- | --- | --- | --- | --- | --- | --- | --- | --- |
|  |  | Complete case | | Imputed | |  | Complete case | | Imputed | |  | Complete case | | Imputed | |
|  |  | Crude OR (95% CI) | p | Crude OR (95% CI) | p |  | Crude OR (95% CI) | p | OR (95% CI) | p |  | Crude OR (95% CI) | p | OR (95% CI) | p |
| Age | Lowest | 1 |  | 1 |  |  | 1 |  | 1 |  |  | **1** |  | 1 |  |
|  | Middle | **1.11 (1.01 – 1.23)** | **0.04** | **1.11 (1.01 – 1.23)** | **0.04** |  | 0.96 (0.83 - 1.11) | 0.565 | 0.96 (0.83 - 1.11) | 0.565 |  | **1.34 (1.16 - 1.55)** | **<0.001** | **1.34 (1.16 - 1.55)** | **<0.001** |
|  | Highest | **1.29 (1.16 – 1.43)** | **<0.001** | **1.29 (1.16 – 1.43)** | **<0.001** |  | 1.11 (0.96 - 1.28) | 0.157 | 1.11 (0.96 - 1.28) | 0.157 |  | **1.52 (1.32 - 1.76)** | **<0.001** | **1.52 (1.32 - 1.76)** | **<0.001** |
| Gender | Male | 1 |  | **1** |  |  | 1 |  | 1 |  |  | 1 |  | **1** |  |
|  | Female | **1.18 (1.07 – 1.30)** | **0.001** | **1.18 (1.07 – 1.30)** | **0.001** |  | **1.19 (1.04 - 1.37)** | **0.012** | **1.19 (1.04 - 1.37)** | **0.012** |  | **1.19 (1.04 - 1.36)** | **0.011** | **1.19 (1.04 - 1.36)** | **0.011** |
| BMI | <25 kg/m^2^ | 1 |  | 1 |  |  | 1 |  | 1 |  |  | 1 |  | 1 |  |
|  | 25 - <30 kg/m^2^ | 0.99 (0.90 – 1.10) | 0.918 | 1.00 (0.91 - 1.10) | 0.999 |  | 0.99 (0.85 - 1.14) | 0.855 | 0.99 (0.87 - 1.13) | 0.89 |  | 1.00 (0.88 - 1.15) | 0.972 | 1.01 (0.89 - 1.16) | 0.818 |
|  | ≥30 kg/m^2^ | 1.04 (0.93 – 1.16) | 0.489 | 1.05 (0.95 – 1.16) | 0.398 |  | 1.14 (0.98 - 1.32) | 0.096 | 1.11 (0.97 - 1.28) | 0.139 |  | 0.95 (0.81 - 1.10) | 0.467 | 0.97 (0.84 - 1.12) | 0.67 |
| Smoking | Never | 1 |  | 1 |  |  | 1 |  | 1 |  |  | 1 |  | 1 |  |
|  | Ever | **1.13 (1.03 – 1.24)** | **0.008** | **1.13 (1.03 – 1.23)** | **0.012** |  | 1.10 (0.97 - 1.25) | 0.141 | 1.06 (0.93 - 1.21) | 0.37 |  | **1.15 (1.02 - 1.30)** | **0.028** | 1.15 (0.99 - 1.33) | 0.066 |
|  | Current | **1.14 (1.02 – 1.27)** | **0.017** | **1.19 (1.07 – 1.32)** | **0.001** |  | 1.14 (0.98 - 1.32) | 0.095 | **1.18 (1.02 - 1.37)** | **0.031** |  | 1.10 (0.95 - 1.27) | 0.21 | **1.20 (1.06 - 1.37)** | **0.004** |
| DAS28 | Lowest | 1 |  | 1 |  |  | **1** |  | 1 |  |  | **1** |  | 1 |  |
|  | Middle | 0.99 (0.89 – 1.10) | 0.833 | 0.99 (0.90 – 1.10) | 0.899 |  | **1.01 (0.87 - 1.17)** | **0.881** | 1.02 (0.88 - 1.18) | 0.798 |  | **0.99 (0.86 - 1.14)** | **0.943** | 0.97 (0.84 - 1.11) | 0.648 |
|  | Highest | **1.21 (1.09 – 1.34)** | **<0.001** | **1.21 (1.09 – 1.33)** | **<0.001** |  | **1.30 (1.13 - 1.50)** | **<0.001** | **1.28 (1.12 - 1.47)** | **<0.001** |  | **1.16 (1.01 - 1.33)** | **0.035** | **1.17 (1.02 - 1.33)** | **0.026** |
| DAS28-P | Lowest | 1 |  | 1 |  |  | 1 |  | 1 |  |  | 1 |  | 1 |  |
|  | Middle | 0.96 (0.87 – 1.07) | 0.483 | 0.96 (0.87 – 1.06) | 0.377 |  | 0.99 (0.86 - 1.15) | 0.94 | 1.00 (0.90 - 1.15) | >0.99 |  | 0.96 (0.83 - 1.10) | 0.535 | 0.97 (0.85 - 1.11) | 0.653 |
|  | Highest | 1.01 (0.91 – 1.12) | 0.936 | 1.01 (0.92 - 1.12) | 0.821 |  | **1.00 (0.87 - 1.16)** | >0.99 | 1.05 (0.91 - 1.21) | 0.521 |  | **0.98 (0.85 - 1.14)** | **0.826** | 1.00 (0.87 - 1.14) | 0.945 |
| ESR | Lowest | **1** |  | 1 |  |  | **1** |  | 1 |  |  | **1** |  | 1 |  |
|  | Middle | **1.00 (0.90 – 1.11)** | **>0.99** | 1.01 (0.92 - 1.12) | 0.798 |  | **0.97 (0.83 - 1.13)** | **0.696** | 0.98 (0.85 - 1.13) | 0.768 |  | **1.05 (0.91 - 1.22)** | **0.505** | 1.03 (0.90 - 1.19) | 0.648 |
|  | Highest | **1.29 (1.16 – 1.43)** | **<0.001** | **1.29 (1.17 – 1.42)** | **<0.001** |  | **1.37 (1.19 - 1.59)** | **<0.001** | **1.36 (1.19 - 1.56)** | **<0.001** |  | **1.25 (1.08 - 1.44)** | **0.002** | **1.24 (1.09 - 1.42)** | **0.002** |
| SJC | Lowest | 1 |  | 1 |  |  | 1 |  | 1 |  |  | 1 |  | 1 |  |
|  | Middle | 0.90 (0.81 – 1.00) | 0.053 | 0.91 (0.83 - 1.02) | 0.102 |  | 0.86 (0.74 - 0.99) | 0.04 | **0.87 (0.76 - 1.01)** | **0.069** |  | 0.90 (0.78 - 1.04) | 0.17 | 0.90 (0.78 - 1.03) | 0.122 |
|  | Highest | 1.03 (0.93 – 1.14) | 0.56 | 1.04 (0.95 - 1.15) | 0.392 |  | 1.05 (0.91 - 1.20) | 0.506 | 1.06 (0.93 - 1.21) | 0.411 |  | 1.03 (0.90 - 1.18) | 0.68 | 1.02 (0.90 - 1.17) | 0.738 |
| VAS-GH | Lowest | **1** |  | 1 |  |  | **1** |  | 1 |  |  | 1 |  | 1 |  |
|  | Middle | **1.01 (0.91 – 1.12)** | **0.915** | 1.04 (0.94 - 1.15) | 0.476 |  | **1.15 (0.99 - 1.33)** | **0.076** | 1.17 (1.02 - 1.36) | 0.031 |  | 0.87 (0.76 - 1.01) | 0.062 | 0.91 (0.79 - 1.04) | 0.163 |
|  | Highest | **1.24 (1.12 – 1.37)** | **<0.001** | **1.25 (1.13 – 1.38)** | **<0.001** |  | **1.42 (1.23 - 1.64)** | **<0.001** | **1.42 (1.24 - 1.64)** | **<0.001** |  | 1.07 (0.93 - 1.27) | 0.344 | 1.11 (0.97 - 1.27) | 0.134 |
| TJC | Lowest | 1 |  | 1 |  |  | **1** |  | 1 |  |  | 1 |  | 1 |  |
|  | Middle | 1.05 (0.95 – 1.17) | 0.345 | 1.05 (0.95 – 1.15) | 0.39 |  | **1.04 (0.90 - 1.21)** | **0.579** | 1.07 (0.92 - 1.23) | 0.386 |  | 1.06 (0.92 - 1.22) | 0.433 | 1.05 (0.91 - 1.20) | 0.511 |
|  | Highest | 1.11 (1.00 – 1.28) | 0.058 | **1.12 (1.01 - 1.24)** | **0.027** |  | **1.17 (1.01 - 1.35)** | **0.04** | **1.20 (1.04 - 1.38)** | **0.011** |  | 1.08 (0.94 - 1.24) | 0.297 | 1.08 (0.94 - 1.23) | 0.31 |
| Duration | Lowest | **1** |  | 1 |  |  | **1** |  | 1 |  |  | **1** |  | 1 |  |
|  | Middle | **1.01 (0.91 – 1.12)** | **0.835** | 1.05 (0.95 - 1.16) | 0.361 |  | **0.87 (0.75 - 1.00)** | **0.047** | 0.88 (0.77 - 1.01) | 0.069 |  | **1.13 (0.98 - 1.31)** | **0.099** | **1.17 (1.01 - 1.34)** | **0.034** |
|  | Highest | **1.12 (1.01 – 1.23)** | **0.037** | **1.14 (1.03 - 1.26)** | **0.009** |  | **0.85 (0.73 - 0.97)** | **0.02** | **0.85 (0.74 - 0.98)** | **0.024** |  | **1.43 (1.24 - 1.64)** | **<0.001** | **1.42 (1.24 - 1.63)** | **<0.001** |
| Serology | Negative | 1 |  | **1** |  |  | 1 |  | **1** |  |  | 1 |  | **1** |  |
|  | Positive | 1.03 (0.95 – 1.12) | 0.465 | 1.03 (0.95 – 1.12) | 0.478 |  | 0.93 (0.82 - 1.04) | 0.203 | 0.93 (0.82 - 1.04) | 0.203 |  | **1.13 (1.00 - 1.27)** | **0.044** | **1.13 (1.00 - 1.27)** | **0.047** |
| Erosions | None | 1 |  | 1 |  |  | 1 |  | 1 |  |  | 1 |  | 1 |  |
|  | Yes | 0.96 (0.89 – 1.05) | 0.384 | 0.96 (0.89 – 1.05) | 0.385 |  | **0.88 (0.79 - 0.99)** | **0.036** | **0.88 (0.79 - 0.99)** | **0.036** |  | 1.04 (0.92 - 1.16) | 0.559 | 1.04 (0.92 - 1.16) | 0.579 |
| Extra-articular | No | 1 |  | 1 |  |  | 1 |  | **1** |  |  | 1 |  | 1 |  |
|  | Yes | **1.24 (1.14 – 1.36)** | **<0.001** | **1.24 (1.14 – 1.36)** | **<0.001** |  | 1.10 (0.97 - 1.25) | 0.144 | 1.10 (0.97 - 1.25) | 0.144 |  | **1.44 (1.28 - 1.62)** | **<0.001** | **1.44 (1.28 - 1.62)** | **<0.001** |
| Co-morbidity | No | 1 |  | **1** |  |  | 1 |  | 1 |  |  | 1 |  | **1** |  |
|  | Yes | **1.26 (1.16 – 1.37)** | **<0.001** | **1.26 (1.16 – 1.37)** | **<0.001** |  | **1.20 (1.07 - 1.35)** | **0.002** | **1.20 (1.07 - 1.35)** | **0.002** |  | **1.33 (1.18 - 1.49)** | **<0.001** | **1.33 (1.18 - 1.49)** | **<0.001** |
| HAQ | Lowest | **1** |  | **1** |  |  | 1 |  | 1 |  |  | 1 |  | 1 |  |
|  | Middle | **0.88 (0.79 – 0.98)** | **0.024** | **0.90 (0.81 - 0.99)** | **0.033** |  | 0.93 (0.80 - 1.07) | 0.31 | 0.94 (0.82 - 1.08) | 0.374 |  | 0.89 (0.77 - 1.03) | 0.118 | 0.90 (0.78 - 1.03) | 0.133 |
|  | Highest | **0.94 (0.84 – 1.04)** | **0.216** | 0.94 (0.85 – 1.03) | 0.198 |  | 0.90 (0.78 - 1.04) | 0.174 | 0.90 (0.79 - 1.04) | 0.161 |  | 0.97 (0.84 - 1.12) | 0.716 | 0.95 (0.83 - 1.09) | 0.511 |
| SF36-Vitality | Best | **1** |  | **1** |  |  | **1** |  | **1** |  |  | 1 |  | 1 |  |
|  | Middle | **1.09 (0.97 – 1.21)** | **0.15** | 1.10 (1.00 - 1.22) | 0.061 |  | **1.17 (1.00 - 1.36)** | **0.059** | **1.17 (1.01 - 1.35)** | **0.045** |  | 1.00 (0.86 - 1.17) | 0.97 | 1.07 (0.93 - 1.23) | 0.352 |
|  | Worst | **1.24 (1.11 – 1.37)** | **<0.001** | **1.21 (1.10 - 1.34)** | **<0.001** |  | **1.43 (1.24 - 1.65)** | **<0.001** | **1.41 (1.23 - 1.62)** | **<0.001** |  | 1.12 (0.98 - 1.29) | 0.11 | **1.15 (1.01 - 1.32)** | **0.033** |
| SF36-Mental Health | Best | 1 |  | 1 |  |  | **1** |  | **1** |  |  | **1** |  | **1** |  |
|  | Middle | **1.25 (1.12 – 1.39)** | **<0.001** | **1.22 (1.10 - 1.35)** | **<0.001** |  | **1.36 (1.17 - 1.58)** | **<0.001** | **1.33 (1.15 - 1.53)** | **<0.001** |  | **1.22 (1.06 - 1.42)** | **0.007** | **1.18 (1.03 - 1.36)** | **0.019** |
|  | Worst | **1.23 (1.11 – 1.36)** | **<0.001** | **1.23 (1.12 - 1.35)** | **<0.001** |  | **1.30 (1.13 - 1.50)** | **<0.001** | **1.30 (1.13 - 1.49)** | **<0.001** |  | **1.18 (1.03 - 1.36)** | **0.017** | **1.18 (1.04 - 1.35)** | **0.014** |
| SF36-Physical Function | Best | 1 |  | 1 |  |  | **1** |  | **1** |  |  | **1** |  | **1** |  |
|  | Middle | **1.22 (1.10 – 1.36)** | **<0.001** | **1.24 (1.12 - 1.37)** | **<0.001** |  | **1.26 (1.08 - 1.46)** | **0.003** | **1.23 (1.107 - 1.42)** | **0.005** |  | **1.21 (1.05 - 1.41)** | **0.011** | **1.33 (1.15 - 1.53)** | **<0.001** |
|  | Worst | **1.68 (1.50 – 1.87)** | **<0.001** | **1.61 (1.46 - 1.78)** | **<0.001** |  | **1.62 (1.40 -- 1.89)** | **<0.001** | **1.52 (1.33 - 1.75)** | **<0.001** |  | **1.77 (1.53 - 2.06)** | **<0.001** | **1.81 (1.58 - 2.07)** | **<0.001** |
| SF36-Bodily Pain | Best | 1 |  | 1 |  |  | **1** |  | **1** |  |  | **1** |  | **1** |  |
|  | Middle | **1.25 (1.12 – 1.39)** | **<0.001** | **1.19 (1.08 - 1.33)** | **0.001** |  | **1.22 (1.04 - 1.44)** | **0.016** | **1.22 (1.05 - 1.42)** | **0.009** |  | **1.24 (1.06 - 1.44)** | **0.006** | **1.22 (1.06 - 1.41)** | **0.005** |
|  | Worst | **1.60 (1.43 – 1.78)** | **<0.001** | **1.51 (1.37 - 1.67)** | **<0.001** |  | **1.82 (1.57 - 2.12)** | **<0.001** | **1.77 (1.53 - 2.03)** | **<0.001** |  | **1.41 (1.22 - 1.64)** | **<0.001** | **1.40 (1.23 - 1.61)** | **<0.001** |
| Steroids | None | 1 |  | 1 |  |  | 1 |  | **1** |  |  | 1 |  | **1** |  |
|  | At baseline | **1.18 (1.08 – 1.28)** | **<0.001** | **1.18 (1.08 – 1.28)** | **<0.001** |  | 1.07 (0.95 - 1.21) | 0.286 | 1.07 (0.95 - 1.21) | 0.286 |  | **1.29 (1.15 - 1.46)** | **<0.001** | **1.29 (1.15 - 1.46)** | **<0.001** |
| Methotrexate | None | 1 |  | 1 |  |  | 1 |  | **1** |  |  | 1 |  | **1** |  |
|  | At baseline | **0.65 (0.59 – 0.71)** | **<0.001** | **0.65 (0.59 – 0.71)** | **<0.001** |  | **0.65 (0.57 - 0.74)** | **<0.001** | **0.65 (0.57 - 0.74)** | **<0.001** |  | **0.55 (0.49 - 0.63)** | **<0.001** | **0.55 (0.49 - 0.63)** | **<0.001** |
| Sulphasalazine | None | 1 |  | 1 |  |  | 1 |  | **1** |  |  | 1 |  | **1** |  |
|  | At baseline | **0.69 (0.61 – 0.77)** | **<0.001** | **0.69 (0.61 – 0.77)** | **<0.001** |  | **0.79 (0.67 - 0.94)** | **0.006** | **0.79 (0.67 - 0.94)** | **0.006** |  | **0.59 (0.49 - 0.70)** | **<0.001** | **0.59 (0.49 - 0.70)** | **<0.001** |
| Lefunomide | None | 1 |  | 1 |  |  | 1 |  | **1** |  |  | 1 |  | **1** |  |
|  | At baseline | **1.28 (1.10 – 1.48)** | **0.002** | **1.28 (1.10 – 1.48)** | **0.002** |  | **1.48 (1.21 - 1.80)** | **<0.001** | **1.48 (1.21 - 1.80)** | **<0.001** |  | 1.20 (0.98 - 1.48) | 0.086 | 1.20 (0.98 - 1.48) | 0.086 |
| Azathioprine | None | 1 |  | 1 |  |  | 1 |  | **1** |  |  | 1 |  | **1** |  |
|  | At baseline | 1.21 (0.92 – 1.59) | 0.193 | 1.21 (0.92 – 1.59) | 0.193 |  | 1.03 (0.69 - 1.55) | 0.834 | 1.03 (0.69 - 1.55) | 0.834 |  | 1.39 (0.97 - 1.98) | 0.073 | 1.39 (0.97 - 1.98) | 0.073 |
| Hydroxychloroquine | None | 1 |  | 1 |  |  | 1 |  | **1** |  |  | 1 |  | **1** |  |
|  | At baseline | **0.76 (0.66 – 0.88)** | **<0.001** | **0.76 (0.66 – 0.88)** | **<0.001** |  | 0.86 (0.70 - 1.05) | 0.16 | 0.86 (0.70 - 1.05) | 0.16 |  | **0.74 (0.60 - 0.92)** | **0.005** | **0.74 (0.60 - 0.92)** | **0.005** |
| Gold | None | 1 |  | 1 |  |  | 1 |  | **1** |  |  | 1 |  | **1** |  |
|  | At baseline | 0.89 (0.64 – 1.24) | 0.558 | 0.89 (0.64 – 1.24) | 0.558 |  | 0.514 (0.16 - 1.66) | 0.35 | 0.514 (0.16 - 1.66) | 0.35 |  | 0.79 (0.31 - 2.03) | 0.824 | 0.79 (0.31 - 2.03) | 0.824 |

Univariate OR (95% CI) for discontinuation of TNF-inhibitors that were started at baseline by 1 year follow up (complete cases and imputed values analyses are include). The p values have not been adjusted for multiple comparisons. Continuous variables divided into tertiles except for body mass index (BMI). For each variable, the highest tertile is that which signifies worst health (including SF36 variables, which have been reversed). ESR = Erythrocyte sedimentation rate, SJC=swollen joint count, TJC=tender joint count, VAS-GH=visual analogue scale-general health. Significant results are highlighted in **bold.**
